# Comparing therapeutic modulators of the SOD1 G93A Amyotrophic Lateral Sclerosis mouse pathophysiology

**DOI:** 10.3389/fnins.2022.1111763

**Published:** 2023-01-19

**Authors:** Albert J. B. Lee, Tyler E. Kittel, Renaid B. Kim, Thao-Nguyen Bach, Tian Zhang, Cassie S. Mitchell

**Affiliations:** ^1^Laboratory for Pathology Dynamics, Biomedical Engineering, Georgia Institute of Technology and Emory University School of Medicine, Atlanta, GA, United States; ^2^Center for Machine Learning, Georgia Institute of Technology, Atlanta, GA, United States; ^3^University of Michigan Medical School, Ann Arbor, MI, United States; ^4^University of Texas at Dallas, Dallas, TX, United States

**Keywords:** Amyotrophic Lateral Sclerosis (ALS), motoneuron disease, neuromuscular, pharmacology, mouse model, SOD1 G93A mouse, SOD1

## Abstract

**Introduction:**

Amyotrophic Lateral Sclerosis (ALS) is a paralyzing, multifactorial neurodegenerative disease with limited therapeutics and no known cure. The study goal was to determine which pathophysiological treatment targets appear most beneficial.

**Methods:**

A big data approach was used to analyze high copy SOD1 G93A experimental data. The secondary data set comprised 227 published studies and 4,296 data points. Treatments were classified by pathophysiological target: apoptosis, axonal transport, cellular chemistry, energetics, neuron excitability, inflammation, oxidative stress, proteomics, or systemic function. Outcome assessment modalities included onset delay, health status (rotarod performance, body weight, grip strength), and survival duration. Pairwise statistical analysis (two-tailed *t*-test with Bonferroni correction) of normalized fold change (treatment/control) assessed significant differences in treatment efficacy. Cohen’s *d* quantified pathophysiological treatment category effect size compared to “all” (e.g., all pathophysiological treatment categories combined).

**Results:**

Inflammation treatments were best at delaying onset (*d* = 0.42, *p* > 0.05). Oxidative stress treatments were significantly better for prolonging survival duration (*d* = 0.18, *p* < 0.05). Excitability treatments were significantly better for prolonging overall health status (*d* = 0.22, *p* < 0.05). However, the absolute best pathophysiological treatment category for prolonging health status varied with disease progression: oxidative stress was best for pre-onset health (*d* = 0.18, *p* > 0.05); excitability was best for prolonging function near onset (*d* = 0.34, *p* < 0.05); inflammation was best for prolonging post-onset function (*d* = 0.24, *p* > 0.05); and apoptosis was best for prolonging end-stage function (*d* = 0.49, *p* > 0.05). Finally, combination treatments simultaneously targeting multiple pathophysiological categories (e.g., polytherapy) performed significantly (*p* < 0.05) better than monotherapies at end-stage.

**Discussion:**

In summary, the most effective pathophysiological treatments change as function of assessment modality and disease progression. Shifting pathophysiological treatment category efficacy with disease progression supports the homeostatic instability theory of ALS disease progression.

## 1. Introduction

Amyotrophic Lateral Sclerosis (ALS) is a fatal motor neuron disease for which there is currently no cure. Presently, only three mainstream therapies exist, which can delay progression or modestly improve survival: riluzole (Fang et al., 2018), which targets neural excitability; edaravone (Abe et al., 2014), which targets oxidative stress; and sodium phenylbutyrate–taurursodiol, which blocks apoptotic pathways in the mitochondria and endoplasmic reticulum (Paganoni et al., 2022).

Most ALS cases are considered sporadic. Only 5–10% of clinical patients have a known familial ALS connection (Renton et al., 2014). About 20% of familial ALS patients have a superoxide dismutase 1 (SOD1) mutation (Trieu et al., 2000). Nucleotide repeat expansions, namely, c9orf72, have also been implicated in ALS progression (Puentes et al., 2021). Recent polygenic assessment identified several environmental risk factors for ALS, such as educational attainment, physical activity, smoking, and tenseness/restlessness (Bandres-Ciga et al., 2019). Prior studies have also proposed the presence or absence of antecedent conditions as an associative ALS risk factor (Mitchell et al., 2015b; Hollinger et al., 2016). Thus, ALS is likely a multifactorial and multi-scalar condition, involving the interplay of several pathogenic pathways and risk factors to initiate and propagate the spread of motor neuron death (Goodall and Morrison, 2006).

Transgenic ALS mice have enabled scientists to examine many facets of the ALS pathology. The widespread availability, reproducibility and characterization of the SOD1 G93A model had made it a foundational tool for ALS research (Pfohl et al., 2015). In fact, the SOD1 G93A model is one of the most widely published transgenic mouse models (Kim et al., 2015). SOD1 G93A is an abbreviation for superoxide dismutase-1 glycine 93 to alanine, which represents the specific mutation type. Hundreds of creative treatments for ALS have been assessed in the SOD1 G93A mouse model, ranging from melatonin (Zhang et al., 2013) to bee venom (Yang et al., 2010).

Nine broad ontological categories of aberrant physiological functions have been defined in SOD1 G93A ALS mice (Mitchell and Lee, 2012a) and used to guide ALS pharmaceutics (Pandya et al., 2013): apoptosis, axonal transport, cellular chemistry, biological energetics, neuron excitability, inflammation, oxidative stress, proteomics, and systemic function. The SOD1 G93A pathophysiological treatment categories are briefly described here; for further details please see Kim et al. (2015). Cellular apoptosis includes programmed cell death or aberrant and/or premature cell death. Axonal transport includes the anterograde transport of essential nutrients, neurofilaments, organelles, or neurotransmitters to the axon and retrograde transport of products for degradation to the soma. Enzymatic cellular chemistry includes enzymes, metal ion balance, and chelators necessary for cellular biochemistry and housekeeping. Energetics includes aerobic and anaerobic cellular respiration and related processes necessary to provide energy to cells and tissues. Neural excitability includes the ability to maintain ion gradients, membrane potential, and execute action potentials required for normal neuron firing. Inflammation includes the role of astrocytes, oligodendrocytes, inflammatory cytokines, and immune mediators to maintain and protect the neural and neuromuscular environments. Oxidative stress includes conversion of free radicals, heat shock proteins, and other processes to prevent DNA or RNA damage. Proteomic function includes translation of required fully functional proteins and degradation of aberrant or misfolded proteins. Systemic function includes intersecting higher-level system functions (neural network communication, muscle function, ambulation, etc.) or other multifaceted functions necessary to maintain overall good physical health.

The goal of this study was to determine which of the nine general SOD1 G93A transgenic ALS mouse pathophysiological treatment targets are most beneficial. Treatment efficacy was assessed as a function of pathophysiological treatment category, outcome assessment modality, and disease stage. Assessment modalities included ALS symptom onset delay, mouse health status (body weight, rotarod performance grip strength, etc.), and survival duration. A data science approach was utilized to assess SOD1 G93A pathophysiological treatment trends from 227 published data sources. The elucidation of trends from such a large pool of data can assist in prioritizing research for future ALS pharmaceutics.

## 2. Materials and methods

The general methodology included: organizing and normalizing previously curated SOD1 G93A ALS mouse data; assigning each experimental treatment to one of nine defined pathophysiological categories; calculating normalized effect size; and statistical analysis to compare the efficacy of each pathophysiological treatment category to the “all” treatments category (e.g., all pathophysiological treatment categories combined).

### 2.1. Data source

This study is a secondary analysis of existing SOD1 G93A experimental data. Data originated from a relational database created and maintained by the Laboratory for Pathology Dynamics at Georgia Institute of Technology. The database contained extracted and curated data on SOD1 G93A transgenic ALS mice studies indexed in PubMed, published through the year 2019, and written in English. The relational database automatically connected to and queried PubMed using the keywords “Amyotrophic Lateral Sclerosis” and “SOD1 G93A” and “transgenic mouse” to identify studies for download, processing and curation. Please see our previously published work (Mitchell et al., 2015a) for further details on the database, including the data structure, human data curation protocol, and quality control protocol.

For the present analysis, the following primary data source attributes were required for inclusion: (1) utilization of high copy B6SJL mice or B6SJL mice backcrossed with genotypes that produced the same ALS disease progression timeline (e.g., equivalent time of onset and average time of death); (2) included results for both an untreated *in vivo* transgenic control group and a treated *in vivo* transgenic group; (3) included treatments were explicitly intended to ameliorate ALS pathology or symptoms; (4) treatment(s) had a defined target that could be mapped to one of the nine defined pathophysiological categories; (5) included a defined and quantified time of onset assessment and survival assessment; (6) included quantitative *in vivo* health status modality measurements spanning at least two of the main three disease stages (pre-onset, onset, and post-onset).

The above criteria resulted in 227 peer-reviewed studies being selected for the present analysis. A full list of primary data source citations can be found in the Supplementary material. Supplementary Table 1 contains citations for unharmful or beneficial treatments included in the primary analysis. Supplementary Table 2 contains the citations to the unintentionally negative (or harmful) treatments, which were analyzed separately.

### 2.2. Pathophysiological treatment category definitions

All treatments were organized into one of the nine previously defined pathophysiological categories (Kim et al., 2015) based on their intended target mechanism to treat ALS mice (Table 1): axonal transport, apoptosis, chemistry, energetics, excitability, inflammation, oxidative stress, proteomics, systemic. Notably, systemic, the largest and most varied category, included cell, diet, exercise, growth factor, gene, transplant, and “other” unconventional or holistic treatments.

**TABLE 1:** Pathophysiological treatment categories, definitions, and examples.

Category	Definition	Examples
Apoptosis	Repair aberrant apoptotic pathways or inhibit pro-apoptotic pathways	zVAD-fmk, AEOL 10150, p75 knockout
Axonal transport	Repair deficit in both anterograde and retrograde axonal transport	Noscapine or anti-NRP1
Chemistry	Repair mishandling of metal or an imbalance in vital nutrients	DP-109, DP-460, Iron chelator VK-28, magnesium pidolate
Energetics	Primarily target mitochondrial dysfunction and use of bioenergy (ATP, glucose)	Uridine, metformin, methimazole
Excitability	Repair or prevent excitotoxic damage through various mechanisms	Riluzole, caffeine, progesterone, cannabinoid
Inflammation	Modulate the inflammation process	Inhibition of transglutaminase 2, rofecoxib
Oxidative stress	Lower the free radicals and oxidative stress levels	Riboflavin and pramipexole
Proteomics	Repair or prevent aberrant protein aggregation.	Guanabenz and resveratrol
Systemic	Considered to have an effect at a systemic level, rather than cellular or subcellular level. Subdivided into: cell therapy, diet, exercise, growth factor, gene therapy, transplant, and unconventional	Stem cell therapy, light therapy, fibroblast growth factors

Treatments for SOD1 G93A ALS transgenic mice were aggregated by their intended mechanism of action or modulation of one of nine pathophysiology types.

### 2.3. Monotherapy versus polytherapy definitions

To meet the objective of comparing treatments from different pathophysiological categories, a nuanced definition of polytherapy was implemented. In this study, polytherapy treatments were defined as treatments that target two or more of the nine pathophysiological treatment categories, whereas monotherapy treatments targeted a single pathophysiological category. This definition was crafted to better assess the differences in normalized treatment effect across categories. Most polytherapies were combinatorial treatment regimens that were applied simultaneously, such as creatine and rofecoxib or celecoxib (Klivenyi et al., 2004). In the rare case when a treatment was known to have explicit effects on multiple pathophysiological categories, it was classified here as a polytherapy. For example, bee venom was found to directly impact both apoptosis and inflammation (Yang et al., 2010), and thus, was classified as a polytherapy. On the other hand, if two treatments of the same pathophysiological category were simultaneously applied, the treatment was classified here as a monotherapy. For example, PRE-084 and resveratrol both ameliorate proteomic damage (Mancuso et al., 2014), and thus, was classified as a monotherapy. However, the vast majority of monotherapies were, in fact, comprised of a single drug or substance targeting a single pathophysiological category.

### 2.4. Disease outcome assessment modality definitions

Quantifiable data points were categorized into three groups of assessment modalities: onset, health status, and survival. Onset indicators included measures like probability of onset, paralysis onset, onset age, etc. Survival assessment included measures like survival duration, survival rate, age at humane exsanguination, cumulative survival, etc. Health status comprised remaining functional metrics such as body weight, rotarod performance, grip strength, and grooming frequency. Only health metrics were temporally assessed throughout all stages of disease progression.

### 2.5. Data normalization for effect size calculation

Given the inherent variability in experimental procedures, metrics, and units, all data was normalized to be on the same scale. Each SOD1 G93A mouse treatment data point was paired with a time-matched untreated SOD1 G93A mouse data point (e.g., control group) from within the same study. Treatment effects were assessed using fold change. The quantified outcome of the treated group was divided by that of the untreated group (e.g., treated SOD1 G93A/untreated SOD1 G93A) to determine the normalized effect. Only treatments that performed equal to the untreated group (normalized effect = 1) or better than the untreated group (normalized effect >1) were included in the primary analysis. Treatments that were meant to improve performance but were unintentionally harmful (e.g., normalized effect <1) were excluded from the primary analysis and assessed separately.

### 2.6. Time bin selection

The binning process provides a detailed assessment of different treatment categories’ effectiveness for each temporal stage in post-natal days. Iterative clustering and dimensional reduction with principal component analysis in Matlab (the Mathworks, Inc., Natick, MA, USA) was performed to produce a scree plot (variance explained versus number of components). The optimal number of bins (seven) was determined using the elbow method to ensure sufficient variance explained and approximately equal bin sample size. The calculated normalized effect sizes were divided into seven bins according to mice post-natal age: 0–70 days, 71–85 days, 86–100 days, 101–110 days, 110–120 days, 121–130 days, and 131 + days.

### 2.7. Statistical analysis

Two methods were jointly utilized to evaluate and compare pathophysiological treatment groups (Sullivan and Feinn, 2012). The joint method: (1) assessed significant differences in normalized treatment effect using 2-sample, 2-tailed *t*-tests and; (2) assessed relative treatment magnitudes utilizing Cohen’s effect size (also known as Cohen’s *d*). To mitigate the bias from any one study and to enable inclusion of studies where mouse group size was not recorded, each included data point was weighted equally regardless of its mouse sample size.

#### 2.7.1. Determination of significant difference in normalized effect

The normalized effect size of each pathophysiological treatment category (explained in “Section 2.5. Data normalization for effect size calculation”) was compared to “all” (e.g., all pathophysiological treatment categories combined). Significant differences between each pathophysiological treatment category and “all” were assessed using a 2-sample, 2-tailed *t*-test in Microsoft Excel. The alpha was set to 0.05. A Bonferroni correction was utilized to adjust the p-value for multiple comparisons.

Note that statistically comparing each pathophysiological category to “all” was deemed more appropriate than individually comparing each pathophysiological category to every other pathophysiological category. Comparing each category individually to every other category would have resulted in 36 pairwise comparisons. Such a high number of comparisons would have inevitably increased the likelihood of Type I or II errors. In contrast, comparing each pathophysiological category to “all” resulted in nine pairwise comparisons–one for each pathophysiological category. Each comparison tests whether the given pathophysiological treatment category performed significantly better or worse than “all.”

#### 2.7.2. Determination of relative magnitude of treatment effect using Cohen’s *d*

The normalized effect sizes represent the effect relative to untreated transgenic control mice, as outlined in (“Section 2.5. Data normalization for effect size calculation”). Cohen’s effect, also known as Cohen’s *d*, is a statistical measure of relative effect size. Cohen’s effect quantifies each pathophysiological category’s relative treatment magnitude compared to “all” (e.g., all pathophysiological treatment categories combined). The typical rule of thumb for Cohen’s *d* effect size is that *d* < 0.2 is a small effect; 0.2 < *d* < 0.5 is a medium effect, and *d* ≥ 0.85 is a large effect (Sullivan and Feinn, 2012). A positive Cohen’s *d* means the individual pathophysiological treatment category performed better than “all,” whereas a negative Cohen’s *d* means the pathophysiological treatment category performed worse than “all.”

## 3. Results

The results of this study include an overview of the data sources utilized; assessment of overall or aggregate treatment effect size; assessment of treatment effect as a function of pathophysiological treatment category, disease stage or time bin, and assessment modality (onset, health status, survival); assessment of negative (harmful) treatments; and assessment of polytherapy versus monotherapy.

### 3.1. Data overview

A total of 227 published data sources were used to analyze beneficial SOD1 G93A ALS mouse treatments. The distribution of published data sources (i.e., journal articles) was as follows: apoptosis (19); axonal transport (3); chemistry (16); energetics (42); excitability (28); inflammation (34); oxidative stress (33); proteomics (18); systemic (75); and all (227). Note that 44 journal articles contained treatments that spanned more than one pathophysiological treatment category (e.g., polytherapy). Polytherapy was analyzed separately from monotherapy.

SOD1 G93A mouse researchers start treatment early to better magnify and elucidate etiological impact. Analysis of post-onset effect size was not possible because less than 5% of ALS preclinical therapies in the curated database (Mitchell et al., 2015a) started after functional symptom onset. The mean treatment start date for presented analysis was 49.8 ± 1.5 days, which was well before high copy SOD1 G93A transgenic ALS mouse symptom onset. No pathophysiological treatment category had a significantly different mean treatment start date (*p* > 0.05, Supplementary Table 3). As such, initial treatment start time was not considered a confounding factor in the present analysis.

The average onset age for untreated SOD1 G93A transgenic mice was 101.3 ± 0.7 days and average time of death was 131.3 ± 3.1 days. These means are nearly identical to previous analysis examining the average symptom onset for mixed gender untreated high copy SOD1 G93A mice (Pfohl et al., 2015). There was no significant difference (*p* > 0.05) in onset or time of death in the untreated mice for each pathophysiological category. As such, differences in untreated control were not considered a confounding factor in the present analysis.

The normalized effect size for untreated control (e.g., untreated SOD1 G93A mice) is always 1. A normalized effect size <1 means that treatment performed worse than untreated ALS mice, and thus, was actually harmful. Here, only unharmful or beneficial treatments (normalized effect sizes ≥1) were included in the primary analysis. Table 2 illustrates the data point counts for beneficial treatment analysis organized by assessment type (health, onset, survival) and time bins for health status measurements (0–70 days, 71–85 days, 86–100 days, 101–110 days, 111–120 days, 121–130 days, 131 + days), and pathophysiological treatment category. Briefly, the number of health status data points was 3,463; survival had 282 data points; and onset age comprised 551 data points. The data point counts by pathophysiological category was as follows: apoptosis (335); axonal transport (32); chemistry (128); energetics (932); excitability (605); inflammation (563); oxidative stress (507); proteomics (242); systemic (952); and all (4,296). Supplementary Figure 1 illustrates the unaggregated data points for all beneficial treatments organized by time bin and pathophysiological treatment category.

**TABLE 2:** SOD1 G93A data counts for consistently beneficial treatments (e.g., normalized effect size ≥1).

Metric	Apoptosis	Axonal transport	Chemistry	Energetics	Excitability	Inflammation	Oxidative stress	Proteomics	Systemic	ALL
Health status	253	19	99	833	543	466	390	144	716	3,463
0–70 day	55	3	27	150	78	44	60	20	142	579
71–85 day	29	4	13	120	96	60	48	15	74	459
86–100 day	49	5	17	163	121	88	56	33	135	667
101–110 day	39	1	9	86	66	85	50	24	120	480
111–120 day	45	4	23	149	133	79	68	30	105	636
121–130 day	18	1	8	72	25	66	64	14	61	329
131 + day	18	1	2	93	24	44	44	8	79	313
Onset	55	7	17	66	38	70	79	63	156	579
Survival	27	6	12	33	24	27	38	35	80	263
TOTAL	335	32	128	932	605	563	507	242	952	4,296

Data is organized by one of nine pathophysiological treatment targets in the column and assessment modality in rows (heath status, onset age, or survival). The seven temporal bins under health status entail health status metrics specifically measured during the corresponding post-natal age periods (in days). Each data point counted represents a normalized effect size calculated using treatment and control data from the same data source (e.g., treated SOD1 G93A/untreated control SOD1 G93A).

### 3.2. Aggregate analysis of all beneficial treatments by pathophysiological category

**FIGURE 1:**
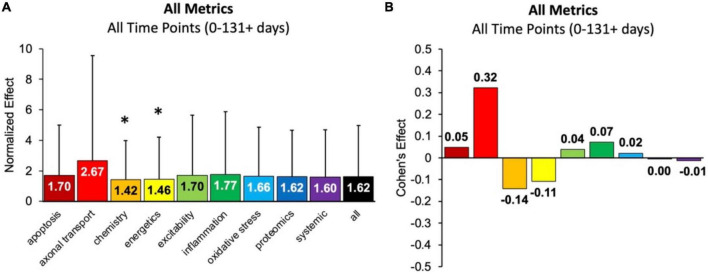
Analysis of pathophysiological treatment category performance aggregated across all mouse age groups (0–131 + days). **(A)** Normalized effect size for each pathophysiological treatment category. Error bars represent standard deviation. Each pathophysiological treatment category was compared to the “all” treatments category (black bar) with * denoting a significant difference (*p* < 0.05). **(B)** Calculated Cohen’s effect size (also known as Cohen’s *d*) for each pathophysiological treatment category. Cohen’s effect is a statistical measure of each pathophysiological treatment category’s effect size relative to the “all” treatments category (e.g., all pathophysiological treatment categories combined).

First, the aggregate (or combined) normalized treatment effect size was separately assessed for treatment category. Figure 1A illustrates the average normalized effect size for each pathophysiological treatment category performance aggregated across all time bins and all assessment modalities. The overall treated average or “all” normalized effect is 1.62. For reference, the untreated control (e.g., untreated SOD1 G93A ALS mice) normalized effect size is always 1. Each pathophysiological category’s normalized effect size (NE) is statistically compared to the “all” treatments category (e.g., all pathophysiological treatment categories combined). Chemistry (NE = 1.42) and energetics (NE = 1.46) were significantly less (*p* < 0.05) than the “all” treatments category.

Figure 1B illustrates the Cohen’s effect for each pathophysiological treatment category’s performance aggregated across all time bins and all assessment modalities. Here Cohen’s effect is the pathophysiological effect size compared to “all.” Notably, axonal transport has both the highest normalized effect (NE = 2.67) and Cohen’s effect (*d* = 0.32). However, its large standard deviation and small sample size decreased statistical power for axonal transport. In general, the Cohen’s *d* effect size shown in Figure 1B represents small effect sizes.

**FIGURE 2:**
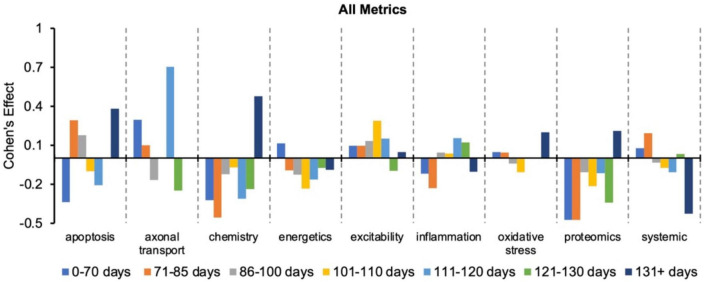
Visualization of how the Cohen’s effect size, *d*, changes over time for each pathophysiological treatment category. No one pathophysiological category consistently performed better than the “all” treatments category (e.g., all pathophysiological treatment categories combined). Note that axonal transport is excluded in a few time bins due to inadequate sample size. Cohen’s effect is a statistical measure of each pathophysiological treatment category’s effect size for the specified time bin relative to the “all” treatments category of the corresponding time bin.

Figure 2 re-plots the Cohen’s *d* to more easily visualize how effect size changes with disease progression (or time bin) for a given pathophysiological category. Every category has both positive and negative Cohen’s effect sizes, *d*, depending on the time bin. Thus, no category always performs better or worse than the “all” treatments category. Nonetheless, excitability has the most frequent positive Cohen’s effect sizes, which means excitability consistently performs better than the “all” treatments category. In fact, only one excitability time bin (121–130 days) had a negative Cohen’s effect size.

### 3.3. Analysis of onset delay: Inflammation treatments perform best

**FIGURE 3:**
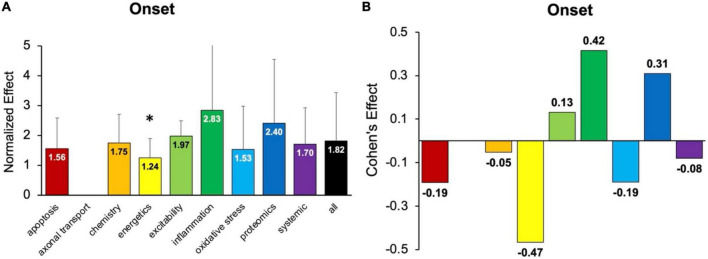
Assessment of pathophysiological treatment category on delaying onset. **(A)** Normalized effect size for onset metrics for each pathophysiological treatment category. Error bars represent standard deviation. Axonal transport was omitted due to inadequate sample size. Each pathophysiological treatment category was compared to the “all” treatments category (black bar) with * denoting a significant difference (*p* < 0.05). **(B)** Calculated Cohen’s effect size for symptom onset for each pathophysiological treatment category. Cohen’s effect is a statistical measure of each pathophysiological treatment category’s effect size on onset relative to “all” treatments.

Onset assessment modalities detect the earliest possible onset symptoms. No pathophysiological treatment category performed significantly better at delaying onset than “all” treatments (e.g., all pathophysiological categories combined) (Figure 3). Energetics treatments performed significantly (*p* < 0.05) worse compared to “all” for delaying onset. However, three categories had small to medium positive Cohen’s *d* effect sizes: inflammation (*d* = 0.42), proteomics (*d* = 0.31), and excitability (*d* = 0.13). Inflammation therapeutics delayed onset by 8.9 ± 1.4 days, which was more than any other pathophysiological treatment category (*p* > 0.05). In summary, inflammation therapeutics were best at delaying onset of symptoms in transgenic high copy SOD1 G93A ALS mice.

### 3.4. Analysis of health status metrics as a function of disease progression

**FIGURE 4:**
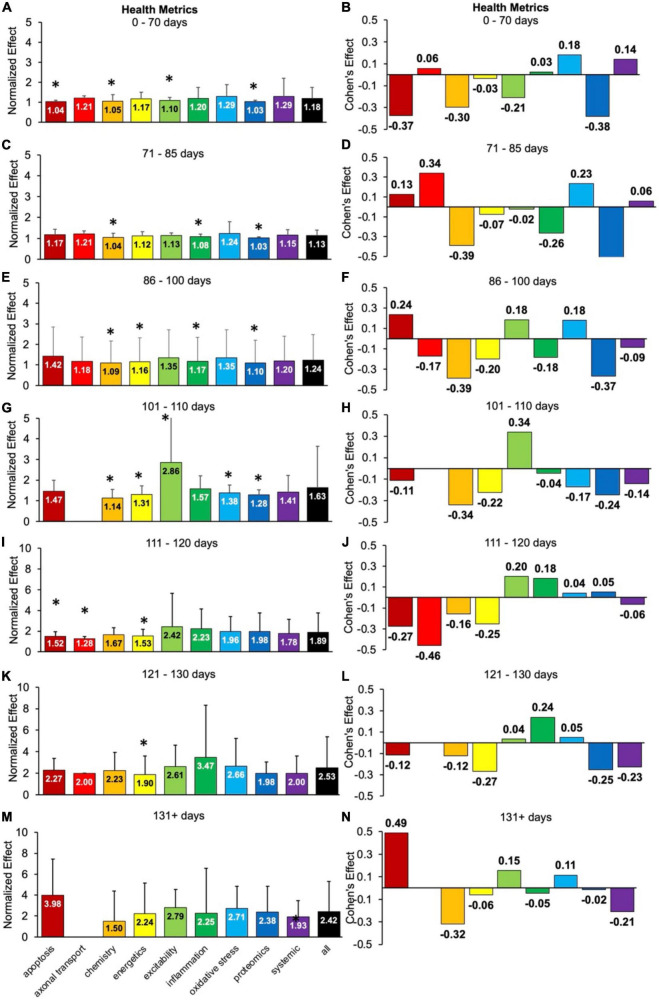
Health status metrics assessed for each pathophysiological treatment category over time (in post-natal days). Normalized effect size is illustrated on the left. Error bars represent standard deviation. Axonal transport is excluded in a few time bins due to inadequate sample size. For each respective time bin, the pathophysiological treatment category was compared to the overall treated average or “all” (black bar) with * denoting a significant difference (*p* < 0.05). Calculated Cohen’s effect size for each pathophysiological treatment category and time bin is illustrated on the right. **(A,B)** 0- 70 days. **(C,D)** 71–85 days. **(E,F)** 86–100 days. **(G,H)** 101–110 days. **(I,J)** 111–120 days. **(K,L)** 121–130 days. **(M,N)** 131 + days.

Health status assessments are typically assessed over the entire life span of SOD1 G93A mice (Figure 4). Health status mostly includes muscle function metrics (e.g., rotarod, grip strength, grooming frequency, etc.), but also includes measures like body weight. The health status time bins were categorized as follows: pre-onset (0–70 days, 71–85 days, 86–100 days), onset (101–110 days), early post-onset (111–120 days), late post-onset (121–130 days) and end-stage (131 + days). In general, the calculated normalized effect sizes are greater in the later disease stages due to health status measurements being more sensitive to post-onset functional decline.

There are clear fluctuations in the best health status treatment performers as a function of disease progression, which are detailed in the subsections below. However, when aggregating over all time bins (not shown), excitability treatments were best at attenuating health status decline (*d* = 0.22, *p* < 0.05).

#### 3.4.1. Oxidative stress therapies perform best for pre-onset health status

Oxidative stress was the overall top performer when assessing health status metrics prior to onset (*p* > 0.05). Oxidative stress had small Cohen’s effect size range of *d* = 0.18 to 0.23 during pre-onset.

For health metrics from 0 to 70 days, no category performed significantly better (*p* > 0.05) than the “all” treatments category (Figure 4A). Apoptosis, chemistry, excitability and proteomics performed significantly worse (*p* < 0.05). The best health status treatments for 0–70 days had small Cohen’s effect sizes: oxidative stress (*d* = 0.18) and systemic (*d* = 0.14) treatments (Figure 4B).

For health metrics from 71 to 85 days, no pathophysiological treatment category performed significantly better (*p* > 0.05) than the “all” treatments category (Figure 4C). However, chemistry, inflammation, and proteomics performed significantly (*p* < 0.05) worse. The best health status performers at 71–85 days were axonal transport (*d* = 0.34) and oxidative stress (*d* = 0.23) treatments, which had moderate Cohen’s effect sizes (Figure 4D).

For health metrics from 86 to 100 days, no pathophysiological treatment category performed significantly better (*p* > 0.05) than the “all” treatments category (Figure 4E). However, chemistry, energetics, inflammation, and proteomics performed significantly (*p* < 0.05) worse. Apoptosis (*d* = 0.24), excitability (*d* = 0.18), and oxidative stress (*d* = 0.18) all had Cohen’s effect sizes that were better than the “all” treatments category (Figure 4F). The tie between excitability and oxidative stress represents the transition from pre-onset (where oxidative stress dominated) to onset (where excitability dominated).

#### 3.4.2. Excitability therapies perform well for onset and early post-onset health status

Once the functional symptoms of onset appear, excitability became the best therapy with a moderate and significant (*p* < 0.05) Cohen’s effect of *d* = 0.34.

For health metrics from 101 to 110 days (e.g., onset period), excitability performed significantly better than the “all” treatment category (*p* < 0.05). However, chemistry, energetics, oxidative stress, and proteomics performed significantly worse than the “all” treatments category (Figure 4G). Excitability also had the largest positive Cohen’s effect size of *d* = 0.34 (Figure 4H). Note there was insufficient data for axonal transport for 101–110 days.

For health metrics from 111 to 120 days (e.g., early post-onset period), no pathophysiological treatment category performed significantly better than the “all” treatments category. However, apoptosis, axonal transport and energetics performed significantly (*p* < 0.05) worse (Figure 4I). Excitability (*d* = 0.20) and inflammation (*d* = 0.18) performed the best (Figure 4J) from 111 to 120 days. The early post-onset time period of 111–120 days illustrates the changing dynamics with disease progression; the impact of excitability treatment has begun to wane compared to its peak Cohen’s *d* at onset. In contrast, the impact of inflammation treatment has begun an upward trajectory.

#### 3.4.3. Inflammation therapies perform best for late post-onset health status

Inflammation was the second-best performer from 111 to 120 days but became the top performer for late post-onset (121–130 days). At late post-onset, the SOD1 G93A mice are experiencing a drastic increase in muscle dysfunction, as measured by rotarod, grip tests, etc.

For health metrics from 121 to 130 days, no pathophysiological treatment category performed significantly better (*p* > 0.05) than the “all” treatments category. However, energetics performed significantly (*p* < 0.05) worse (Figure 4K). The best health status performer for 121–130 days was inflammation, which had a Cohen’s effect size of *d* = 0.24 (Figure 4L).

#### 3.4.4. Apoptosis therapies perform best for end-stage health status

Any data from the timespoints at which the untreated high copy SOD1 G93A mice would normally die or require humane exsanguination were classified in the last time bin (131 + days).

For health metrics from 131 + days, no pathophysiological treatment category performs significantly better or worse (*p* > 0.05) than the “all” treatments category (Figure 4M). The best health status performers for 131 + days based on Cohen’s effect size were: apoptosis (*d* = 0.49), excitability (*d* = 0.15), and oxidative stress (*d* = 0.11) treatments (Figure 4N). Notably, the apoptosis treatments Cohen’s effect size at 131 + days was the largest Cohen’s effect size of all the temporal health status assessments in Figure 4.

### 3.5. Analysis of survival duration: Oxidative stress treatment perform best

Survival metrics mostly encompass measurements of natural survival or time point of required humane exsanguination of transgenic SOD1 G93A ALS mice. In general, survival assessments predominantly correspond to end-stage (131 + days).

**FIGURE 5:**
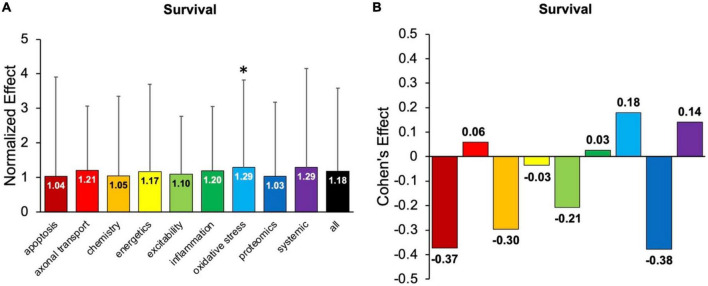
Assessment of pathophysiological treatment category on survival. **(A)** Normalized effect size for survival for each pathophysiological treatment category. Error bars represent standard deviation. Each pathophysiological treatment category was compared to the “all” treatments category (black bar) with * denoting a significant difference (*p* < 0.05). **(B)** Calculated Cohen’s effect size of survival for each pathophysiological treatment category. Cohen’s effect is a statistical measure of each pathophysiological treatment category’s effect size on survival relative to “all” treatments (e.g., all pathophysiological treatment categories combined).

Figure 5 examines the impact of each pathophysiological treatment on survival. Oxidative stress performed significantly (*p* < 0.05) better than the “all” treatments category (Figure 5A) and had a Cohen’s effect of *d* = 0.18 (Figure 5B). Systemic treatments were the second-best performer with a Cohen’s effect size of *d* = 0.14. However, systemic treatments were not significantly (*p* < 0.05) better than the “all” treatments category, likely due to variance (Figure 5A). In summary, oxidative stress therapeutics were the best at improving survival duration in transgenic high copy SOD1 G93A ALS mice.

### 3.6. Unintentional negative (harmful) treatments

**FIGURE 6:**
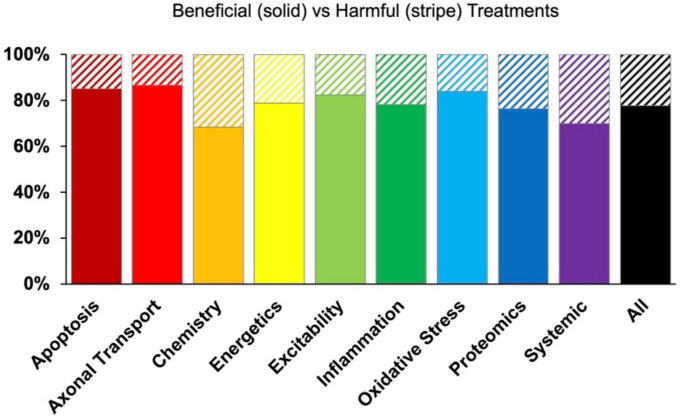
Percent composition of beneficial (solid bars) versus harmful (striped bars) treatments by pathophysiological treatment category. While some treatment categories reported negative results more frequently than others, there was no significant differences (*p* > 0.05).

Despite the intention to ameliorate pathology, some therapies did have an unintended negative functional outcome. Negative treatments (normalized treatment effect size <1) comprised 1,624 data points that were analyzed separately. Due to smaller sample sizes, all assessment modalities (onset, health status, survival) and time bins were combined to examine potential differences in the percentage of harmful treatments between pathophysiological categories. No significant relationships were found that indicated one pathophysiological treatment category was significantly more harmful or had a higher frequency of negative outcomes compared to another (Figure 6).

While there were no global trends of consistently negative treatments, some specific examples were identified. One such consistently negative treatment was caloric restriction, which was assigned to the energetics pathophysiological treatment category. Caloric restriction hypothetically increases mitochondrial oxidative capacity. However, increasing mitochondrial oxidative capacity could be only observed in female SOD1 G93A mice and not males (Patel et al., 2010). Moreover, 65.4% of results after caloric restriction treatment had <1 normalized values across all time stages (Patel et al., 2010).

### 3.7. Polytherapy outperforms monotherapy after onset

**FIGURE 7:**
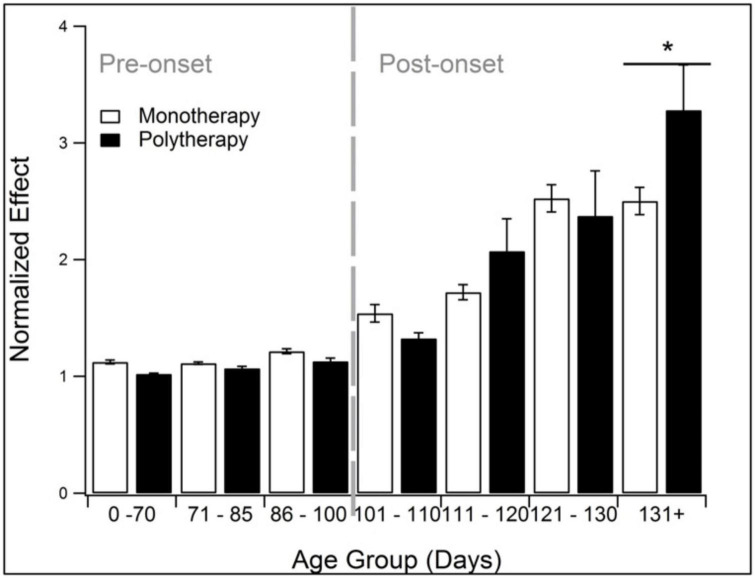
Comparison of the treatment effects of monotherapy and polytherapy. Polytherapy significantly (*p* < 0.05) outperformed monotherapy monotherapy at end stage (131 + days). *Represents *p* < 0.05.

Polytherapy and monotherapy is compared in Figure 7. Polytherapy is the use of multiple treatments, also known as combination therapy or polypharmacy. As noted in the methods, polytherapy was specifically defined here as treatments that target more than one pathophysiological category. There was insufficient sample size to divide the analysis further into discrete pathophysiological categories or assessment modalities. Polypharmacy performed similarly to monotherapy except at end-stage (131 + days). At 131 + days, polytherapy performed significantly better than monotherapy (*p* < 0.05).

## 4. Discussion

Analysis of 4,296 SOD1 G93A transgenic ALS mouse data points enabled assessment of nine pathophysiological treatment categories as a function of assessment modality and disease progression. Below we summarize the study results with context to the published literature.

### 4.1. “Best” treatment fluctuates with progression and assessment modality

**FIGURE 8:**
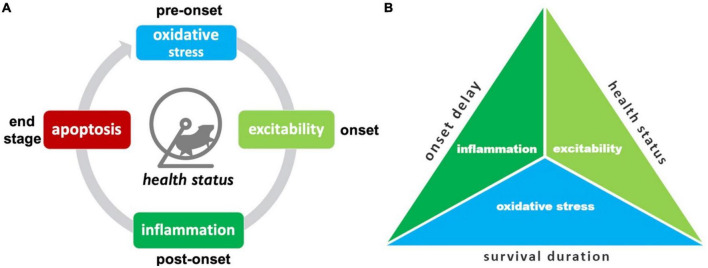
Summary of top SOD1 G93A pathophysiological treatment category performers. **(A)** The “best” pathophysiological category for maintaining health status (rotarod, grip strength, body weight, etc.) changes with disease progression. **(B)** Triad illustrating the best overall treatment performers based on three different assessment modalities. Oxidative stress treatments best delay onset. Excitability treatments were best overall for maintaining health status and/or delaying functional decline. Oxidative stress treatments were best for prolonging survival duration.

Here the “best” treatments are summarized, including a pictogram for easy visualization (Figure 8).

The top treatment performers for reducing health status and functional impairments (e.g., rotarod, grip strength, body weight, etc.) changed with disease progression. While there are fluctuations in treatment efficacy between time bins, there are some clearly denoted health status treatment trends (Figure 8A). Oxidative stress therapies performed best for pre-onset. Excitability treatments were best at prolonging function immediately after onset. Inflammation treatments excelled at maintaining function during late post-onset. Finally, apoptosis therapies excelled most at end-stage.

Examining different assessment modalities (onset delay, health status, survival duration) illustrated differences in top pathophysiological treatment performers (Figure 8B). Inflammation treatments performed best at delaying onset. Excitability treatments performed best overall for slowing functional decline measured by health status modalities across all stages. Oxidative stress most improved survival duration.

### 4.2. Triad of top treatment performers

The triad of consistent top performers are discussed with context to literature.

#### 4.2.1. Inflammation

The inflammation pathophysiological treatment category was a member of the top performers’ triad. In the present analysis, inflammation performed best for attenuating health status decline in the post-onset period and delaying ALS onset.

Inflammatory treatments have been a staple in ALS research for quite awhile (Turner and Talbot, 2008). It has been suggested that very early anti-inflammatory treatment is necessary to interrupt ALS-induced neuroinflammation (Jeyachandran et al., 2015). Besides playing a major role in the pathogenesis of motor neuron death in ALS, neuroinflammation accelerates disease progression (Pandya et al., 2013). Astrocyte-mediated uptake of glutamate, growth factors like VGF (Jordan et al., 2018), tumor necrosis factor (TNF), transglutaminase 2 (TG2), and mSOD1 are also targets of interest associated with neuroinflammation (Oono et al., 2014). Many of the pathways for inflammation and excitability intersect at the astrocytes and microglia, which assist in clearing of neurotransmitters as well activating the nervous system immune response (Jeyachandran et al., 2015).

#### 4.2.2. Oxidative stress

The oxidative stress pathophysiological treatment category was another member of the top performers’ triad. In the present study, oxidative stress performed best for improving pre-onset health status and for prolonging survival duration.

Prior work identified a key change in oxidative stress levels right before symptom onset in SOD1 G93A ALS mouse (Irvin et al., 2015). It appears that innate compensatory mechanisms, namely, antioxidants, increase right before SOD1 G93A ALS symptom onset. This trend is seen again in the present work. Oxidative stress is tied to a series of altered processes in the SOD1 G93A model, including depression of mitochondrial activity in cytochrome C, Cox, and Complex 1 (Nalbandian et al., 2015). Preincubation with oxidant trapping molecules or addition of antioxidants was successful *in vitro* SOD1 G93A models in delaying onset of motoneuron degeneration (Liu et al., 2002).

As is known in clinical ALS, improving end-stage function does not perfectly correlate with survival. Likewise, in ALS mice, the best category for prolonging survival (oxidative stress) differed than the best category for improving end-stage health status or function (apoptosis). Oxidative stress treatment significantly outperformed the overall treated average for improving survival duration. This finding supports prior analysis that found oxidative stress therapies increased SOD1 G93A ALS mouse survival by 11.2% (Bond et al., 2018). Oxidative stress has long been thought to be important in ALS treatment. Supplements like vitamin E (Bond et al., 2020) and the prescription drug for ALS, edaravone (Abe et al., 2014), are used to combat oxidative stress even in late-stage clinical ALS.

#### 4.2.3. Excitability

The excitability pathophysiological treatment category was the third member of the top performers’ triad. Excitability-based treatments were the best overall performer for attenuating health status functional decline. In particular, excitability performed best at onset and during early post-onset.

Glutamate-induced excitotoxicity resulting in motor neuron death was one of the first hypothesized pathogenic mechanisms in ALS (Eisen, 1995; Louvel et al., 1997). As mutant SOD1 (mSOD1) increases the sensitivity of the AMPA receptor to glutamatergic stimulation and disrupts mitochondrial function, affecting the surrounding astrocytes (Van Damme et al., 2003). Most excitability treatments interrupt glutamatergic transmission and lower glutamate concentration, resulting in protection against motor neuron degeneration (Pandya et al., 2013).

Electrophysiology experiments indicate SOD1 G93A mice have changes in excitability with age. Hypoexcitability is seen early and precedes degeneration (Martinez-Silva et al., 2018; Durand et al., 2021; Fogarty, 2021). Changes in motor neuron gains may be to blame (Martinez-Silva et al., 2018; Huh et al., 2021); such results provide support for the hypervigilant regulation hypothesis where the homeostatic instability in ALS mice may be exacerbated by hypervigilant regulatory processes that attempt to compensate but instead over-correct (Mitchell and Lee, 2012a; Irvin et al., 2015). Ironically, excitotoxicity cell death was initially considered to be a later phenomenon in mutant SOD1-mediated neurodegeneration (Ghadge et al., 2003; Van Damme et al., 2003; Rothstein et al., 2005; Ganel et al., 2006; Turner and Talbot, 2008). The present study’s results suggest impact of excitability-based treated closely follows the onset of functional ALS symptoms and are relatively more impactful on health status metrics (Figure 4) than onset or survival. As a specific example, ceftriaxone significantly delayed the decrease in the body weight and muscle strength (Turner and Talbot, 2008).

### 4.3. “Promising” pathophysiological treatments

Two categories, apoptosis and axonal transport, showed promising trends even if they were not consistently top performers.

#### 4.3.1. Apoptosis

Apoptosis treatments appear promising in attenuating SOD1 G93A mouse health status decline at end-stage (131 + days). As such, apoptosis treatments may prove fruitful for improving clinical ALS end-stage quality of life. Other researchers have also observed that agents which block apoptosis in ALS mice primarily affect disease endpoint (Turner and Talbot, 2008). Example anti-apoptotic treatments that were evaluated in late disease include zVAD-fmk, an enzymatic caspase inhibitor (Li et al., 2000), AEOL 10150, a manganese porphyrin with anti-apoptotic properties (Petri et al., 2006), p75 knockout (Kust et al., 2003), and melatonin (Zhang et al., 2013). Apoptosis is the consensus end point for neural degeneration. As such, it is not surprising that apoptosis therapies most impact health status functional decline at end-stage. The most recent United States Food and Drug Administration approved drug for clinical ALS, sodium phenylbutyrate–taurursodiol, blocks apoptotic pathways (Paganoni et al., 2022).

#### 4.3.2. Axonal transport

Available axonal transport treatment sample size was a key limitation for in-depth assessment in the present study. Nonetheless, axonal transport treatments illustrated promise. When aggregating across all time bins and assessment modalities, axonal transport had the largest Cohen’s effect size (Figure 1). Axonal transport treatments also performed well for pre-onset health status at 71–85 days. Axonal transport has been previously shown to be compromised very early in the ALS mouse lifespan, around 20–50 days (Bilsland et al., 2010; Marinkovic et al., 2012). While there is evidence of early axonal transports deficits, there is conflicting results as to whether transport deficits initiate or coincide with the early stages of axon degeneration and retraction (Marinkovic et al., 2012). While axonal transport treatments have been promising (Fanara et al., 2007; Venkova et al., 2014), their precise mechanisms of action remain unclear. Computational work has shown that some neurodegenerative diseases can be differentiated based on their axonal transport profiles (Mitchell and Lee, 2012b). In summary, the promise illustrated by the small pool of axonal transport treatments warrants further research.

### 4.4. Relative underperformers

When examining only the normalized effect sizes for each assessment modality, every pathophysiological treatment category had some measurably positive effect. However, chemistry, energetics, proteomics, and systemic tended to relatively underperform compared to the “all” treatments category. In fact, in several instances these categories performed significantly worse. For chemistry and systemic, the breadth of included treatment mechanisms likely contributed to higher variance. The efficacy of energetics appeared strongly tied to the sign of modulation. For example, caloric restriction was found to be consistently harmful in SOD1 G93A mice (Patel et al., 2010). Similarly, having a lower body mass index has been clinically shown to be more associated with clinical ALS (Mitchell et al., 2015b; Hollinger et al., 2016).

### 4.5. Homeostatic instability to explain why “best” treatments change with progression

The presented SOD1 G93A ALS mouse treatment trends illustrate fluctuations or “oscillations” in treatment efficacy as a function of disease progression and assessment modality. In engineered systems, the presence of measured oscillations is a symptom of aggressive system control (Riahi, 2002). When oscillations become too large, the system becomes increasingly unstable until catastrophic system failure ensues. Likewise, biological systems must exert multifactorial control to maintain homeostasis - the critical balance required to sustain normal function and support life (Billman, 2020). When biological control cannot be properly maintained, growing oscillations result in homeostatic instability that cause pathological symptoms (Mitchell and Lee, 2008). For a recent physiology review further explaining the history, role, and principles of homeostasis in health and disease, please see Billman (2020).

An example where natural oscillations initiate neuropathology is absence epilepsy (Gillary and Niebur, 2016). The neocortex must be able to respond quickly; as such, the network dynamics are very near the edge of stability. Neocortex networks that are very close to instability develop oscillations in the delta range (1–4 Hz), which can cause absence epilepsy (Gillary and Niebur, 2016). Parkinsonian tremors (Mcauley and Marsden, 2000) and secondary spinal cord injury have also shown signs of unstable oscillatory dynamics (Mitchell and Lee, 2008) as part of their respective pathologies.

Likewise, oscillations due to failed homeostasis or “homeostatic instability” has been proposed as a hypothesis for ALS progression (Irvin et al., 2015; Mitchell et al., 2015b; Hollinger et al., 2016). Prior work has demonstrated pervasive oscillating relationships (Mitchell and Lee, 2012a) across multiple pathophysiological categories. For example, natural antioxidant products shoot up prior to ALS onset in SOD1 G93A mice - a likely compensatory mechanism to re-establish stable homeostasis (Irvin et al., 2015; Bond et al., 2018). Similarly, SOD1 G93A transgenic mouse motoneurons oscillate between hyperexcitability and hypoexcitability; these changes in excitability dynamics correlated to different ALS disease stages (Martinez-Silva et al., 2018).

It has been hypothesized that homeostatic instability could be the primary mechanism by which different pathophysiological factors could lead to ALS. That is, it is possible that multiple different perturbations with very different causes (whether sporadic or familial mutation, environmental exposure, or a regulatory change in a given pathophysiological target) could result in motoneuron system instability that is the ALS phenotype (Irvin et al., 2015; Hollinger et al., 2016). In particular, motoneurons are more prone to destabilizing dynamics due to their long axons. Very small changes can be magnified and propagated due to the sprawling orders of magnitude in temporal signaling from soma to synapse. For example, axon potentials are on the order of a millisecond and slow axonal transport is on the order of days to weeks (Mitchell and Lee, 2012b).

The cause of homeostatic instability may be tied to hypervigilant regulation. The statistical observation that clinical ALS patients are otherwise healthier than age- and gender-matched controls was initially used to construct a possible hypervigilant regulation theory (Mitchell et al., 2015b; Hollinger et al., 2016). The hypervigilant regulation theory states that ALS progression results from innately overzealous regulatory control that increases the likelihood of motoneuron system instability (Irvin et al., 2015; Hollinger et al., 2016). Hypervigilant regulation may result in excellent early life health (e.g., less antecedent disease like hypertension, diabetes, etc.) due to the tight regulatory control. However, hypervigilant regulation could contribute to later instability that results in more severe disease (Mitchell et al., 2015b; Hollinger et al., 2016).

Recent experimental ALS mouse electrophysiological studies illustrated that ALS motoneurons do, in fact, illustrate excessive homeostatic gain that results in oscillatory instability (Kuo et al., 2020). “Gain” is a control theory term that determines the aggressiveness to which a controller responds to a change in input (Billman, 2020). If the gain is excessive, the controller over-corrects, resulting in oscillations in the input variable. Likewise, if a neuron’s gain is too high (i.e., hypervigilant), it will cause the neuron to exhibit unstable oscillatory behavior in response to a large perturbation in input (Irvin et al., 2015; Mitchell et al., 2015b; Hollinger et al., 2016). This phenomenon has been seen in the spinal motoneuron properties of young adult SOD1 G93A transgenic mice (Huh et al., 2021).

While the present study cannot confirm the presence of homeostatic instability, the vast changes in pathophysiological treatment category efficacy over time do provide associative support for the hypothesis. The fact that some treatments meant to be beneficial became harmful during some time points (Figure 6) is another possible clue. If homeostatic instability is driving ALS progression, treatments must be timed to appropriately align with the peak or valley of the pathological oscillation(s) in order to be optimally beneficial. As such, treating the overall system instability using a combination of factors (e.g., polypharmacy) may be more helpful than treating the initial perturbation of a single factor.

### 4.6. Polypharmacy for ALS

Polypharmacy may be one cutting-edge technique to better address the complex nature of ALS (Papa et al., 2022), including homeostatic instability (Mitchell and Lee, 2012a). Despite the overall smaller sample size, polytherapy was significantly more beneficial than monotherapy during end-stage in high copy SOD1 G93A mice (Figure 7). Improved therapeutic efficacy in end-stage is important for increasing late-stage quality of life and survival. Previous preclinical and clinical literature demonstrated combination treatments have a synergistic effect: rasagiline and riluzole (Waibel et al., 2004); minocycline and creatine (Klivenyi et al., 2004); Lithium and Neu2000 (Shin et al., 2007); minocycline + riluzole + nimodipine (Kriz et al., 2003); minocycline-creatine and celoxicob-creatine (Gordon et al., 2008); and most recently, riluzole, edaravone, and Nu-9 (Genc et al., 2022). A previous computational study also illustrated that combination therapies may be able to re-stabilize ALS and halt progression by returning the pathophysiology to homeostasis (Mitchell and Lee, 2012a; Irvin et al., 2015). While polypharmacy is an interesting concept moving forward, care must be taken to consider possible drug interactions (Genc et al., 2022).

### 4.7. Limitations

First, pathophysiological treatment ontology aggregation (Kim et al., 2015) was a necessity to ascertain global trends but prevented looking at specific mechanisms. Second, despite aggregation, overall sample size was limited for axonal transport and polytherapy. Third, ALS mouse treatments were initiated pre-onset, whereas clinical ALS patients do not receive treatment until after functional symptom onset. Fourth, there is a high degree of variance and lower precision of common assessment modalities for ALS transgenic disease progression is early disease stages (Weydt et al., 2003). Finally, the SOD1 G93A ALS transgenic mouse model is helpful for elucidating etiology and testing treatments. However, it is not known whether the transgenic animal model fully generalizes to sporadic ALS or only emulates ALS due to familial superoxide dismutase mutations (Louvel et al., 1997).

## 5. Conclusion

The efficacy of SOD1 G93A ALS treatments varies as a function of pathophysiological treatment category, measured assessment modality and temporal disease progression (Figure 8). The triad of overall best treatment performers for each assessment modality was: inflammation (for onset delay), excitability (for attenuating overall health status decline), and oxidative stress treatments (improving survival duration). The absolute best treatments for health status varied with disease progression: oxidative stress (pre-onset), excitability (near onset), inflammation (post-onset), and apoptosis (end-stage). The overall large normalized effect size of axonal transport treatments showed promise, but additional future sample size is necessary to affirm. Despite limited sample size, polytherapy significantly outperformed monotherapy at end-stage. The fluctuations in best treatment with disease progression and superior efficacy of polytherapy at end-stage provide support for the homeostatic instability theory for ALS progression. That is, the dynamic perturbation of multiple different pathophysiological categories could be explained by a pathological system-level instability that thwarts normal homeostatic function.

## Data availability statement

The original contributions presented in this study are included in this article/Supplementary material, further inquiries can be directed to the corresponding author.

## Ethics statement

Ethical review and approval was not required for the animal study because only previously published, publicly available animal model data was utilized for this study.

## Author contributions

AL, TK, RK, and CM: conceptualization. AL, TK, RK, TZ, and CM: methodology and formal analysis. AL, TK, and CM: validation. CM: investigation, resources, writing—review and editing, supervision, project administration, and funding acquisition. AL, TK, RK, TZ, and T-NB: data curation. T-NB and CM: data visualization. AL, TK, and CM: writing—original draft preparation. All authors contributed to the article and approved the submitted version.
