# Supplementary material for: Comparing therapeutic modulators of the SOD1 G93A Amyotrophic Lateral Sclerosis mouse pathophysiology
Source: Front Neurosci. 2023 Jan 19;16:1111763. doi: 10.3389/fnins.2022.1111763 (PMC9893287; doi:10.3389/fnins.2022.1111763)
Supplement: Supplementary file 1 [file Data_Sheet_1.pdf]

Supplementary Material

Comparing therapeutic modulators of the SOD1 G93A Amyotrophic Lateral Sclerosis mouse pathophysiology

1 Supplementary Tables

Supplementary Table 1. Primary article data source citations for positive (beneficial) treatments with a normalized treatment effect size ζ1.

Treatment Category	Source Article Citation
apoptosis	(Li et al., 2000;Kust et al., 2003;Groeneveld et al., 2004;Kaspar et al., 2005;Ryu et al., 2005;Gould et al., 2006;Petri et al., 2006b;Locatelli et al., 2007;Martin et al., 2007;Ohta et al., 2008;Yang et al., 2010;Calvo et al., 2011;Ahn et al., 2012;Gifondorwa et al., 2012;Katsumata et al., 2012;Moreno-Igoa et al., 2012;Takata et al., 2013;Zhang et al., 2013;Pieper et al., 2014)
axonal transport	(Fanara et al., 2007;Perez-Garcia and Burden, 2012;Venkova et al., 2014)
chemistry	(Groeneveld et al., 2003;Pamphlett et al., 2003;Ermilova et al., 2005;Petri et al., 2007;Fornai et al., 2008;Tokuda et al., 2008;Zhang et al., 2008;Kim et al., 2009;Kupershmidt et al., 2009;Barbeito et al., 2010;Guo et al., 2011;Wang et al., 2011;Gianforcaro and Hamadeh, 2012;Gianforcaro et al., 2013;Tokuda et al., 2013;Lee et al., 2015)
energetics	(Matthews et al., 1998;Klivenyi et al., 1999;Mohajeri et al., 1999;Andreassen et al., 2000;Andreassen et al., 2001a;Andreassen et al., 2001b;Derave et al., 2003;Klivenyi et al., 2004;Holzbaur et al., 2006;Miller et al., 2006;Petri et al., 2006a;Zhao et al., 2006;Bordet et al., 2007;Grignaschi et al., 2007;Koh et al., 2007a;Koh et al., 2007b;Messi et al., 2007;Wang et al., 2007a;Kieran et al., 2008;Martinez et al., 2008;Zhang et al., 2008;Choi et al., 2009;Morrison et al., 2009;Amante et al., 2010;Bigini et al., 2011;Calvo et al., 2011;Kaneb et al., 2011;Venerosi et al., 2011;Garbuzova-Davis et al., 2012;Li et al., 2012a;Li et al., 2012b;Miquel et al., 2012;Moreno-

excitability	Igoa et al., 2012;Saenger et al., 2012;Zhao et al., 2012;Li et al., 2013;Ari et al., 2014;Cacabelos et al., 2014;Miquel et al., 2014;Ono et al., 2014;Emde et al., 2015;Rabinovich-Toidman et al., 2015) (Gurney et al., 1996;Gurney, 1997;Canton et al., 2001;Turner et al., 2003;Van Damme et al., 2003;Raman et al., 2004;Weydt et al., 2005;Fanara et al., 2007;Gifondorwa et al., 2007;Habisch et al., 2007b;Joo et al., 2007;Boston-Howes et al., 2008;Del Signore et al., 2009;Lee et al., 2009;Sekiya et al., 2009;Ghoddoussi et al., 2010;Lincecum et al., 2010;Bame et al., 2012;Kong et al., 2012;Mancuso et al., 2012;Shin et al., 2012;Cervetto et al., 2013;Gerber et al., 2013;Gotkine et al., 2013;Kim et al., 2013;Knippenberg et al., 2013;Potenza et al., 2013;Battaglia et al., 2015)
inflammation	(Olsen et al., 2001;Drachman et al., 2002;Van Den Bosch et al., 2002;Zhu et al., 2002;Pompl et al., 2003;Klivenyi et al., 2004;West et al., 2004;Azari et al., 2005;Kiaei et al., 2005b;Kiaei et al., 2005c;Schutz et al., 2005;Gowing et al., 2006;Kiaei et al., 2006;Yan et al., 2006;Dewil et al., 2007;Fanara et al., 2007;Beers et al., 2008;Neymotin et al., 2009;Gros-Louis et al., 2010;Lincecum et al., 2010;Yang et al., 2010;Keller et al., 2011;Tada et al., 2011;Vaknin et al., 2011;Yang et al., 2011;Dibaj et al., 2012;Kong et al., 2012;Shin et al., 2012;Trumbull et al., 2012;Zhang et al., 2014;Ayers et al., 2015;Lee et al., 2015;Esmaeili et al., 2016;Jeon et al., 2016)
oxidative stress	(Gurney et al., 1996;Hottinger et al., 1997;Barneoud and Curet, 1999;Facchinetti et al., 1999;Klivenyi et al., 1999;Jiang et al., 2000;Andreassen et al., 2001a;Andreassen et al., 2001b;Ferrante et al., 2001;Cudkowicz et al., 2002;Derave et al., 2003;Wu et al., 2003;Klivenyi et al., 2004;West et al., 2004;Crow et al., 2005;Kiaei et al., 2005a;Danzeisen et al., 2006;Koh et al., 2006;Petri et al., 2006b;Weishaupt et al., 2006;Xu et al., 2006;Shin et al., 2007;Chang et al., 2008;Martinez et al., 2008;Choi et al., 2009;Moges et al., 2009;Sekiya et al., 2009;Chen et al., 2010;Zhao et al., 2011;Kong et al., 2012;Shin et al., 2012;Mead et al., 2013;Esmaeili et al., 2016)
proteomics	(Trieu et al., 2000;Kieran et al., 2004;Lorenzl et al., 2006;Gifondorwa et al., 2007;Glas et al., 2007;Sharp et al., 2008;Del Signore et al., 2009;Yang et al., 2009;Markert et al., 2010;Shimazawa et al., 2010;Han et al., 2012;Rabinovich-Toidman et al., 2012;Jiang et al., 2014;Mancuso et al., 2014a;Mancuso et al., 2014b;Song et al., 2014;Tonges et al., 2014;Zhao et al., 2015)
systemic	(Mohajeri et al., 1999;Chen and Ende, 2000;Bordet et al., 2001;Willing et al., 2001;Acsadi et al., 2002;Dreibelbis et al., 2002;Wang et al., 2002;Azari et al., 2003;Garbuzova-Davis et al., 2003;Kirkinezos et al., 2003;Veldink et al., 2003;Corti et al., 2004;Liebetanz et al., 2004;Mahoney et al., 2004;Zheng et al., 2004;Day et al., 2005;Hamadeh et al., 2005;Kaspar et al., 2005;Beers et al., 2006;Garbuzova-Davis et al., 2006;Hamadeh and Tarnopolsky, 2006;Solomon et al., 2006;Corti et al., 2007;Fanara et al., 2007;Habisch et al., 2007a;Martin and Liu, 2007;Mattson et al., 2007;Wang et al., 2007b;Zhao et al., 2007;Ciriza et al., 2008;Garbuzova-Davis et al., 2008;McCrate and Kaspar, 2008;Stam et al., 2008;Chiu et al., 2009;Crochemore et al., 2009;Moges et al., 2009;Poulletier de Gannes et al., 2009;Wu et al., 2009;Carreras et al., 2010;Dodge et al., 2010;Hadano et al., 2010;Kim et al., 2010;Moreno-Igoa et al., 2010;Patel et al., 2010;Shimazawa et al., 2010;Turner et al., 2010;Bigini et al., 2011;Tada et al., 2011;Towne et al., 2011;Bame et al., 2012;Feng et al., 2012;Fontanilla et al., 2012;Garbuzova-Davis et al., 2012;Gerber et al., 2012;Knippenberg et al., 2012a;Knippenberg et al., 2012b;Lee et al., 2012;Uccelli et al., 2012;Foust et al., 2013;Zhou et al., 2013;Ayers et al., 2015;Battaglia et al., 2015;Chen et al., 2015;Chung et al., 2015;Emde et al., 2015;Kelly et al., 2015;Lee et al., 2015;Li et al., 2015;Nagahara et al., 2015;Ng et al., 2015;Rabinovich-Toidman et al., 2015;Vieira et al., 2015;Zhao et al., 2015;Esmaeili et al., 2016;Jeon et al., 2016)

Supplementary Table 2. Sub-analysis article data source citations for negative treatments with a normalized treatment effect size < 1 (e.g. SOD1 G93A treated / SOD1 G93A untreated control). These citations correspond to the data utilized to construct Figure 6 in the main article.

Treatment Category	Source Article Citation
apoptosis	(Li et al., 2000;Kust et al., 2003;Groeneveld et al., 2004;Kaspar et al., 2005;Ryu et al., 2005;Petri et al., 2006b;Martin et al., 2007;Ohta et al., 2008;Yang et al., 2010;Calvo et al., 2011;Gifondorwa et al., 2012;Katsumata et al., 2012;Moreno-Igoa et al., 2012;Zhang et al., 2013)
axonal transport	(Fanara et al., 2007;Venkova et al., 2014)
chemistry	(Groeneveld et al., 2003;Pamphlett et al., 2003;Ermilova et al., 2005;Petri et al., 2007;Zhang et al., 2008;Kupershmidt et al., 2009;Gianforcaro and Hamadeh, 2012;Gianforcaro et al., 2013;Lee et al., 2015)
energetics	(Klivenyi et al., 1999;Mohajeri et al., 1999;Andreassen et al., 2001a;Andreassen et al., 2001b;Derave et al., 2003;Klivenyi et al., 2004;Holzbaur et al., 2006;Miller et al., 2006;Petri et al., 2006a;Bordet et al., 2007;Grignaschi et al., 2007;Koh et al., 2007a;Messi et al., 2007;Wang et al., 2007a;Martinez et al., 2008;Zhang et al., 2008;Choi et al., 2009;Morrison et al., 2009;Calvo et al., 2011;Kaneb et al., 2011;Venerosi et al., 2011;Li et al., 2012a;Li et al., 2012b;Miquel et al., 2012;Moreno-Igoa et al., 2012;Saenger et al., 2012;Zhao et al., 2012;Li et al., 2013;Ari et al., 2014;Cacabelos et al., 2014;Xie et al., 2015)
excitability	(Gurney et al., 1996;Canton et al., 2001;Raman et al., 2004;Fanara et al., 2007;Gifondorwa et al., 2007;Habisch et al., 2007b;Joo et al., 2007;Del Signore et al., 2009;Lee et al., 2009;Sekiya et al., 2009;Lincecum et al., 2010;Bame et al., 2012;Kong et al., 2012;Shin et al., 2012;Cervetto et al., 2013;Gerber et al., 2013;Kim et al., 2013;Knippenberg et al., 2013;Potenza et al., 2013;Battaglia et al., 2015)
inflammation	(Drachman et al., 2002;Van Den Bosch et al., 2002;Pompl et al., 2003;Klivenyi et al., 2004;West et al., 2004;Azari et al., 2005;Kiaei et al., 2005c;Gowing et al., 2006;Kiaei et al., 2006;Yan et al., 2006;Fanara et al., 2007;Lincecum et al., 2010;Yang et al., 2010;Keller et al., 2011;Tada et al., 2011;Vaknin et al., 2011;Yang et al., 2011;Zhang et al., 2011;Dibaj et al., 2012;Kong et al., 2012;Shin et al., 2012;Zhang et al., 2014;Ayers et al., 2015;Lee et al., 2015)
oxidative stress	(Gurney et al., 1996;Barneoud and Curet, 1999;Facchinetti et al., 1999;Klivenyi et al., 1999;Andreassen et al., 2001a;Andreassen et al., 2001b;Ferrante et al., 2001;Cudkowicz et al., 2002;Derave et al., 2003;Wu et al., 2003;Klivenyi et al., 2004;West et al., 2004;Crow et al., 2005;Danzeisen et al., 2006;Petri et al., 2006b;Weishaupt et al., 2006;Shin et al., 2007;Chang et al., 2008;Martinez et al., 2008;Choi et al., 2009;Moges et al., 2009;Sekiya et al., 2009;Chen et al., 2010;Zhao et al., 2011;Shin et al., 2012;Mead et al., 2013)
proteomics	(Kieran et al., 2004;Gifondorwa et al., 2007;Sharp et al., 2008;Del Signore et al., 2009;Yang et al., 2009;Markert et al., 2010;Han et al., 2012;Rabinovich-Toidman et al., 2012;Jiang et al., 2014;Mancuso et al., 2014a;Mancuso et al., 2014b;Tonges et al., 2014;Zhao et al., 2015)
systemic	(Mohajeri et al., 1999;Chen and Ende, 2000;Bordet et al., 2001;Willing et al., 2001;Acsadi et al., 2002;Dreibelbis et al., 2002;Wang et al., 2002;Azari et al., 2003;Kirkinezos et al., 2003;Azzouz et al., 2004;Corti et al., 2004;Liebetanz et al., 2004;Mahoney et al., 2004;Day et al., 2005;Hamadeh et al., 2005;Kaspar et al., 2005;Beers et al., 2006;Garbuzova-Davis et al., 2006;Hamadeh and Tarnopolsky, 2006;Solomon et al., 2006;Habisch et al., 2007a;Garbuzova-Davis et al., 2008;Stam et al., 2008;Chiu et al., 2009;Crochemore et al., 2009;Moges et al., 2009;Poulletier de Gannes et al., 2009;Wu et al., 2009;Carreras et al., 2010;Dodge et al., 2010;Hadano et al., 2010;Kim et al., 2010;Moreno-Igoa et al., 2010;Patel et al., 2010;Bigini et al., 2011;Towne et al., 2011;Bame et al., 2012;Fontanilla et al., 2012;Knippenberg et al., 2012a;Knippenberg et al., 2012b;Lee et al., 2012;Moreno-Igoa et al., 2012;Uccelli et al., 2012;Foust et al., 2013;Ayers et al., 2015;Battaglia et al., 2015;Kelly et al., 2015;Lee et al., 2015;Li et al., 2015;Ng et al., 2015;Vieira et al., 2015;Xie et al., 2015;Zhao et al., 2015)

Supplementary Table 3. Treatment start date for each pathophysiological category. Differences are in days ± standard error (± SE). There were no significant differences between treatment categories (p>0.05).

**Category**	**apop-tosis**	**axon transport**	**chem-istry**	**ener-getics**	**excit-ability**	**inflam-mation**	**oxidative stress**	**prote-omics**	**systemic**	**all**
mean treatment start day	44.1 ± 8.8	52.2 ± 11.5	50.9 ± 7.5	45.0 ± 6.5	53.2 ± 4.5	54.2 ± 3.8	48.4 ± 4.7	41.7 ± 5.1	48.6 ± 4.1	49.5 ± 1.8

2 Supplementary Figures

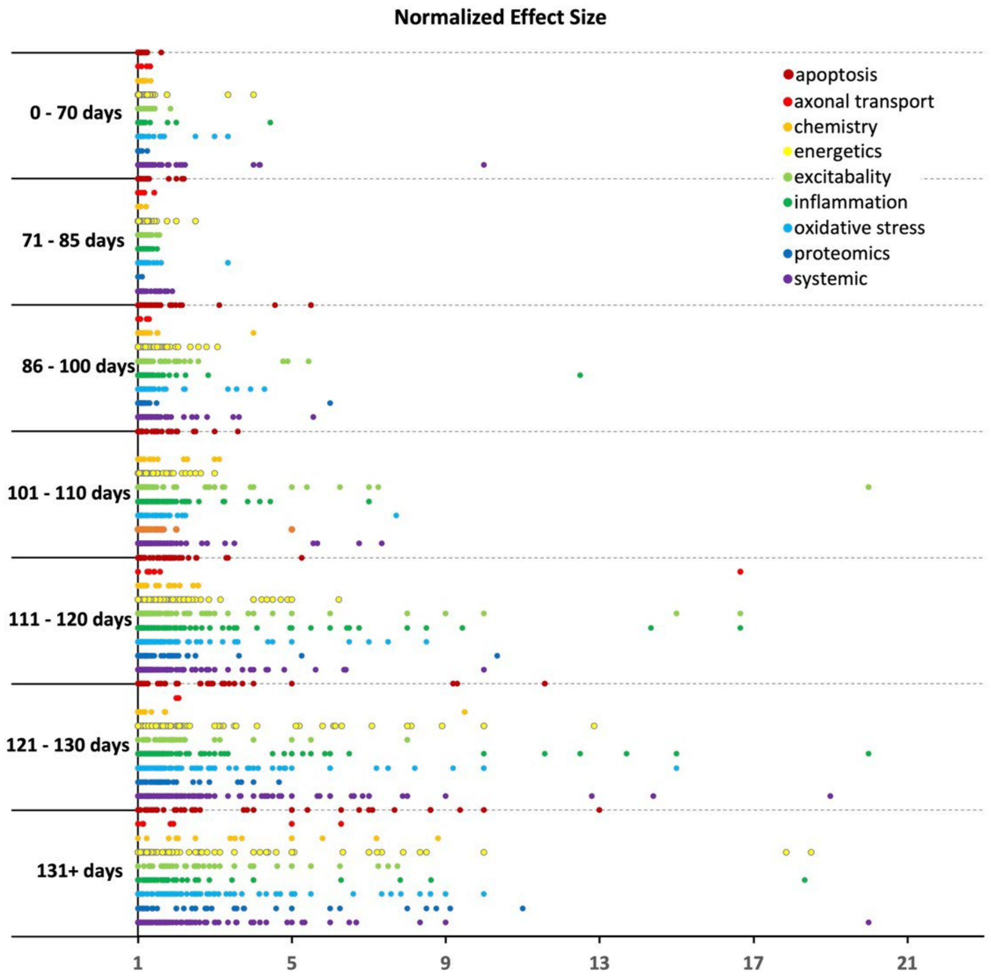

Supplementary Figure 1. Visual comparison of SOD1 G93A transgenic mouse normalized treatment effect values for the primary analysis (normalized effect [illegible] 1). The x-axis corresponds to the normalized effect size (treated SOD1 G93A / untreated SOD1 G93A). The y-axis categories correspond to the mouse age group. The colors correspond to the pathophysiological treatment categories shown in the legend. The normalized effect sizes increased in later time bins due to the exaggerated functional differences in health status between treated and untreated SOD1 G93A ALS mice.

3 Supplementary References

Acsadi, G., Anguelov, R.A., Yang, H., Toth, G., Thomas, R., Jani, A., Wang, Y., Ianakova, E., Mohammad, S., Lewis, R.A., and Shy, M.E. (2002). Increased survival and function of SOD1 mice after glial cell-derived neurotrophic factor gene therapy. *Hum Gene Ther* 13**,** 1047-1059.

Ahn, S.W., Kim, J.E., Park, K.S., Choi, W.J., Hong, Y.H., Kim, S.M., Kim, S.H., Lee, K.W., and Sung, J.J. (2012). The neuroprotective effect of the GSK-3beta inhibitor and influence on the extrinsic apoptosis in the ALS transgenic mice. *J Neurol Sci* 320**,** 1-5.

Amante, D.J., Kim, J., Carreiro, S.T., Cooper, A.C., Jones, S.W., Li, T., Moody, J.P., Edgerly, C.K., Bordiuk, O.L., Cormier, K., Smith, K., Ferrante, R.J., and Rusche, J. (2010). Uridine ameliorates the pathological phenotype in transgenic G93A-ALS mice. *Amyotroph Lateral Scler* 11**,** 520-530.

Andreassen, O.A., Dedeoglu, A., Friedlich, A., Ferrante, K.L., Hughes, D., Szabo, C., and Beal, M.F. (2001a). Effects of an inhibitor of poly(ADP-ribose) polymerase, desmethylselegiline, trientine, and lipoic acid in transgenic ALS mice. *Exp Neurol* 168**,** 419-424.

Andreassen, O.A., Dedeoglu, A., Klivenyi, P., Beal, M.F., and Bush, A.I. (2000). N-acetyl-L-cysteine improves survival and preserves motor performance in an animal model of familial amyotrophic lateral sclerosis. *Neuroreport* 11**,** 2491-2493.

Andreassen, O.A., Jenkins, B.G., Dedeoglu, A., Ferrante, K.L., Bogdanov, M.B., Kaddurah-Daouk, R., and Beal, M.F. (2001b). Increases in cortical glutamate concentrations in transgenic amyotrophic lateral sclerosis mice are attenuated by creatine supplementation. *J Neurochem* 77**,** 383-390.

Ari, C., Poff, A.M., Held, H.E., Landon, C.S., Goldhagen, C.R., Mavromates, N., and D'agostino, D.P. (2014). Metabolic therapy with Deanna Protocol supplementation delays disease progression and extends survival in amyotrophic lateral sclerosis (ALS) mouse model. *PLoS One* 9**,** e103526.

Ayers, J.I., Fromholt, S., Sinyavskaya, O., Siemienski, Z., Rosario, A.M., Li, A., Crosby, K.W., Cruz, P.E., Dinunno, N.M., Janus, C., Ceballos-Diaz, C., Borchelt, D.R., Golde, T.E., Chakrabarty, P., and Levites, Y. (2015). Widespread and efficient transduction of spinal cord and brain following neonatal AAV injection and potential disease modifying effect in ALS mice. *Mol Ther* 23**,** 53-62.

Azari, M.F., Lopes, E.C., Stubna, C., Turner, B.J., Zang, D., Nicola, N.A., Kurek, J.B., and Cheema, S.S. (2003). Behavioural and anatomical effects of systemically administered leukemia inhibitory factor in the SOD1(G93A G1H) mouse model of familial amyotrophic lateral sclerosis. *Brain Res* 982**,** 92-97.

Azari, M.F., Profyris, C., Le Grande, M.R., Lopes, E.C., Hirst, J., Petratos, S., and Cheema, S.S. (2005). Effects of intraperitoneal injection of Rofecoxib in a mouse model of ALS. *Eur J Neurol* 12**,** 357-364.

Azzouz, M., Ralph, G.S., Storkebaum, E., Walmsley, L.E., Mitrophanous, K.A., Kingsman, S.M., Carmeliet, P., and Mazarakis, N.D. (2004). VEGF delivery with retrogradely transported lentivector prolongs survival in a mouse ALS model. *Nature* 429**,** 413-417.

Bame, M., Pentiak, P.A., Needleman, R., and Brusilow, W.S. (2012). Effect of sex on lifespan, disease progression, and the response to methionine sulfoximine in the SOD1 G93A mouse model for ALS. *Gend Med* 9**,** 524-535.

Barbeito, A.G., Martinez-Palma, L., Vargas, M.R., Pehar, M., Manay, N., Beckman, J.S., Barbeito, L., and Cassina, P. (2010). Lead exposure stimulates VEGF expression in the spinal cord and extends survival in a mouse model of ALS. *Neurobiol Dis* 37**,** 574-580.

Barneoud, P., and Curet, O. (1999). Beneficial effects of lysine acetylsalicylate, a soluble salt of aspirin, on motor performance in a transgenic model of amyotrophic lateral sclerosis. *Exp Neurol* 155**,** 243-251.

Battaglia, G., Riozzi, B., Bucci, D., Di Menna, L., Molinaro, G., Pallottino, S., Nicoletti, F., and Bruno, V. (2015). Activation of mGlu3 metabotropic glutamate receptors enhances GDNF and GLT-1 formation in the spinal cord and rescues motor neurons in the SOD-1 mouse model of amyotrophic lateral sclerosis. *Neurobiol Dis* 74**,** 126-136.

Beers, D.R., Henkel, J.S., Xiao, Q., Zhao, W., Wang, J., Yen, A.A., Siklos, L., Mckercher, S.R., and Appel, S.H. (2006). Wild-type microglia extend survival in PU.1 knockout mice with familial amyotrophic lateral sclerosis. *Proc Natl Acad Sci U S A* 103**,** 16021-16026.

Beers, D.R., Henkel, J.S., Zhao, W., Wang, J., and Appel, S.H. (2008). CD4+ T cells support glial neuroprotection, slow disease progression, and modify glial morphology in an animal model of inherited ALS. *Proc Natl Acad Sci U S A* 105**,** 15558-15563.

Bigini, P., Veglianese, P., Andriolo, G., Cova, L., Grignaschi, G., Caron, I., Daleno, C., Barbera, S., Ottolina, A., Calzarossa, C., Lazzari, L., Mennini, T., Bendotti, C., and Silani, V. (2011). Intracerebroventricular administration of human umbilical cord blood cells delays disease progression in two murine models of motor neuron degeneration. *Rejuvenation Res* 14**,** 623-639.

Bordet, T., Buisson, B., Michaud, M., Drouot, C., Galea, P., Delaage, P., Akentieva, N.P., Evers, A.S., Covey, D.F., Ostuni, M.A., Lacapere, J.J., Massaad, C., Schumacher, M., Steidl, E.M., Maux, D., Delaage, M., Henderson, C.E., and Pruss, R.M. (2007). Identification and characterization of cholest-4-en-3-one, oxime (TRO19622), a novel drug candidate for amyotrophic lateral sclerosis. *J Pharmacol Exp Ther* 322**,** 709-720.

Bordet, T., Lesbordes, J.C., Rouhani, S., Castelnau-Ptakhine, L., Schmalbruch, H., Haase, G., and Kahn, A. (2001). Protective effects of cardiotrophin-1 adenoviral gene transfer on neuromuscular degeneration in transgenic ALS mice. *Hum Mol Genet* 10**,** 1925-1933.

Boston-Howes, W., Williams, E.O., Bogush, A., Scolere, M., Pasinelli, P., and Trotti, D. (2008). Nordihydroguaiaretic acid increases glutamate uptake in vitro and in vivo: therapeutic implications for amyotrophic lateral sclerosis. *Exp Neurol* 213**,** 229-237.

Cacabelos, D., Ayala, V., Ramirez-Nunez, O., Granado-Serrano, A.B., Boada, J., Serrano, J.C., Cabre, R., Nadal-Rey, G., Bellmunt, M.J., Ferrer, I., Pamplona, R., and Portero-Otin, M. (2014). Dietary lipid unsaturation influences survival and oxidative modifications of an amyotrophic lateral sclerosis model in a gender-specific manner. *Neuromolecular Med* 16**,** 669-685.

Calvo, A.C., Moreno-Igoa, M., Mancuso, R., Manzano, R., Olivan, S., Munoz, M.J., Penas, C., Zaragoza, P., Navarro, X., and Osta, R. (2011). Lack of a synergistic effect of a non-viral ALS gene therapy based on BDNF and a TTC fusion molecule. *Orphanet J Rare Dis* 6**,** 10.

Canton, T., Bohme, G.A., Boireau, A., Bordier, F., Mignani, S., Jimonet, P., Jahn, G., Alavijeh, M., Stygall, J., Roberts, S., Brealey, C., Vuilhorgne, M., Debono, M.W., Le Guern, S., Laville, M., Briet, D., Roux, M., Stutzmann, J.M., and Pratt, J. (2001). RPR 119990, a novel alpha-amino-3-hydroxy-5-methyl-4-isoxazolepropionic acid antagonist: synthesis, pharmacological properties, and activity in an animal model of amyotrophic lateral sclerosis. *J Pharmacol Exp Ther* 299**,** 314-322.

Carreras, I., Yuruker, S., Aytan, N., Hossain, L., Choi, J.K., Jenkins, B.G., Kowall, N.W., and Dedeoglu, A. (2010). Moderate exercise delays the motor performance decline in a transgenic model of ALS. *Brain Res* 1313**,** 192-201.

Cervetto, C., Frattaroli, D., Maura, G., and Marcoli, M. (2013). Motor neuron dysfunction in a mouse model of ALS: gender-dependent effect of P2X7 antagonism. *Toxicology* 311**,** 69-77.

Chang, Y., Kong, Q., Shan, X., Tian, G., Ilieva, H., Cleveland, D.W., Rothstein, J.D., Borchelt, D.R., Wong, P.C., and Lin, C.L. (2008). Messenger RNA oxidation occurs early in disease pathogenesis and promotes motor neuron degeneration in ALS. *PLoS One* 3**,** e2849.

Chen, K., Northington, F.J., and Martin, L.J. (2010). Inducible nitric oxide synthase is present in motor neuron mitochondria and Schwann cells and contributes to disease mechanisms in ALS mice. *Brain Struct Funct* 214**,** 219-234.

Chen, R., and Ende, N. (2000). The potential for the use of mononuclear cells from human umbilical cord blood in the treatment of amyotrophic lateral sclerosis in SOD1 mice. *J Med* 31**,** 21-30.

Chen, S., Zhang, X.J., Li, L.X., Wang, Y., Zhong, R.J., and Le, W. (2015). Histone deacetylase 6 delays motor neuron degeneration by ameliorating the autophagic flux defect in a transgenic mouse model of amyotrophic lateral sclerosis. *Neurosci Bull* 31**,** 459-468.

Chiu, I.M., Phatnani, H., Kuligowski, M., Tapia, J.C., Carrasco, M.A., Zhang, M., Maniatis, T., and Carroll, M.C. (2009). Activation of innate and humoral immunity in the peripheral nervous system of ALS transgenic mice. *Proc Natl Acad Sci U S A* 106**,** 20960-20965.

Choi, J.K., Kustermann, E., Dedeoglu, A., and Jenkins, B.G. (2009). Magnetic resonance spectroscopy of regional brain metabolite markers in FALS mice and the effects of dietary creatine supplementation. *Eur J Neurosci* 30**,** 2143-2150.

Chung, J.Y., Kim, H.J., and Kim, M. (2015). The protective effect of growth hormone on Cu/Zn superoxide dismutase-mutant motor neurons. *BMC Neurosci* 16**,** 1.

Ciriza, J., Moreno-Igoa, M., Calvo, A.C., Yague, G., Palacio, J., Miana-Mena, F.J., Munoz, M.J., Zaragoza, P., Brulet, P., and Osta, R. (2008). A genetic fusion GDNF-C fragment of tetanus toxin prolongs survival in a symptomatic mouse ALS model. *Restor Neurol Neurosci* 26**,** 459-465.

Corti, S., Locatelli, F., Donadoni, C., Guglieri, M., Papadimitriou, D., Strazzer, S., Del Bo, R., and Comi, G.P. (2004). Wild-type bone marrow cells ameliorate the phenotype of SOD1-G93A ALS mice and contribute to CNS, heart and skeletal muscle tissues. *Brain* 127**,** 2518-2532.

Corti, S., Locatelli, F., Papadimitriou, D., Del Bo, R., Nizzardo, M., Nardini, M., Donadoni, C., Salani, S., Fortunato, F., Strazzer, S., Bresolin, N., and Comi, G.P. (2007). Neural stem cells LewisX+ CXCR4+ modify disease progression in an amyotrophic lateral sclerosis model. *Brain* 130**,** 1289-1305.

Crochemore, C., Virgili, M., Bonamassa, B., Canistro, D., Pena-Altamira, E., Paolini, M., and Contestabile, A. (2009). Long-term dietary administration of valproic acid does not affect, while retinoic acid decreases, the lifespan of G93A mice, a model for amyotrophic lateral sclerosis. *Muscle Nerve* 39**,** 548-552.

Crow, J.P., Calingasan, N.Y., Chen, J., Hill, J.L., and Beal, M.F. (2005). Manganese porphyrin given at symptom onset markedly extends survival of ALS mice. *Ann Neurol* 58**,** 258-265.

Cudkowicz, M.E., Pastusza, K.A., Sapp, P.C., Mathews, R.K., Leahy, J., Pasinelli, P., Francis, J.W., Jiang, D., Andersen, J.K., and Brown, R.H., Jr. (2002). Survival in transgenic ALS mice does not vary with CNS glutathione peroxidase activity. *Neurology* 59**,** 729-734.

Danzeisen, R., Schwalenstoecker, B., Gillardon, F., Buerger, E., Krzykalla, V., Klinder, K., Schild, L., Hengerer, B., Ludolph, A.C., Dorner-Ciossek, C., and Kussmaul, L. (2006). Targeted antioxidative and neuroprotective properties of the dopamine agonist pramipexole and its nondopaminergic enantiomer SND919CL2x [(+)2-amino-4,5,6,7-tetrahydro-6-Lpropylamino-benzathiazole dihydrochloride]. *J Pharmacol Exp Ther* 316**,** 189-199.

Day, W.A., Koishi, K., Nukuda, H., and Mclennan, I.S. (2005). Transforming growth factor-beta 2 causes an acute improvement in the motor performance of transgenic ALS mice. *Neurobiol Dis* 19**,** 323-330.

Del Signore, S.J., Amante, D.J., Kim, J., Stack, E.C., Goodrich, S., Cormier, K., Smith, K., Cudkowicz, M.E., and Ferrante, R.J. (2009). Combined riluzole and sodium phenylbutyrate therapy in transgenic amyotrophic lateral sclerosis mice. *Amyotroph Lateral Scler* 10**,** 85-94.

Derave, W., Van Den Bosch, L., Lemmens, G., Eijnde, B.O., Robberecht, W., and Hespel, P. (2003). Skeletal muscle properties in a transgenic mouse model for amyotrophic lateral sclerosis: effects of creatine treatment. *Neurobiol Dis* 13**,** 264-272.

Dewil, M., Dela Cruz, V.F., Van Den Bosch, L., and Robberecht, W. (2007). Inhibition of p38 mitogen activated protein kinase activation and mutant SOD1(G93A)-induced motor neuron death. *Neurobiol Dis* 26**,** 332-341.

Dibaj, P., Zschuntzsch, J., Steffens, H., Scheffel, J., Goricke, B., Weishaupt, J.H., Le Meur, K., Kirchhoff, F., Hanisch, U.K., Schomburg, E.D., and Neusch, C. (2012). Influence of methylene blue on microglia-induced inflammation and motor neuron degeneration in the SOD1(G93A) model for ALS. *PLoS One* 7**,** e43963.

Dodge, J.C., Treleaven, C.M., Fidler, J.A., Hester, M., Haidet, A., Handy, C., Rao, M., Eagle, A., Matthews, J.C., Taksir, T.V., Cheng, S.H., Shihabuddin, L.S., and Kaspar, B.K. (2010). AAV4-mediated expression of IGF-1 and VEGF within cellular components of the ventricular system improves survival outcome in familial ALS mice. *Mol Ther* 18**,** 2075-2084.

Drachman, D.B., Frank, K., Dykes-Hoberg, M., Teismann, P., Almer, G., Przedborski, S., and Rothstein, J.D. (2002). Cyclooxygenase 2 inhibition protects motor neurons and prolongs survival in a transgenic mouse model of ALS. *Ann Neurol* 52**,** 771-778.

Dreibelbis, J.E., Brown, R.H., Jr., Pastuszak, K.A., Smith, E.R., Kaplan, P.L., and Cudkowicz, M.E. (2002). Disease course unaltered by a single intracisternal injection of BMP-7 in ALS mice. *Muscle Nerve* 25**,** 122-123.

Emde, A., Eitan, C., Liou, L.L., Libby, R.T., Rivkin, N., Magen, I., Reichenstein, I., Oppenheim, H., Eilam, R., Silvestroni, A., Alajajian, B., Ben-Dov, I.Z., Aebischer, J., Savidor, A., Levin, Y., Sons, R., Hammond, S.M., Ravits, J.M., Moller, T., and Hornstein, E. (2015). Dysregulated miRNA biogenesis downstream of cellular stress and ALS-causing mutations: a new mechanism for ALS. *EMBO J* 34**,** 2633-2651.

Ermilova, I.P., Ermilov, V.B., Levy, M., Ho, E., Pereira, C., and Beckman, J.S. (2005). Protection by dietary zinc in ALS mutant G93A SOD transgenic mice. *Neurosci Lett* 379**,** 42-46.

Esmaeili, M.A., Yadav, S., Gupta, R.K., Waggoner, G.R., Deloach, A., Calingasan, N.Y., Beal, M.F., and Kiaei, M. (2016). Preferential PPAR-alpha activation reduces neuroinflammation, and blocks neurodegeneration in vivo. *Hum Mol Genet* 25**,** 317-327.

Facchinetti, F., Sasaki, M., Cutting, F.B., Zhai, P., Macdonald, J.E., Reif, D., Beal, M.F., Huang, P.L., Dawson, T.M., Gurney, M.E., and Dawson, V.L. (1999). Lack of involvement of neuronal nitric oxide synthase in the pathogenesis of a transgenic mouse model of familial amyotrophic lateral sclerosis. *Neuroscience* 90**,** 1483-1492.

Fanara, P., Banerjee, J., Hueck, R.V., Harper, M.R., Awada, M., Turner, H., Husted, K.H., Brandt, R., and Hellerstein, M.K. (2007). Stabilization of hyperdynamic microtubules is neuroprotective in amyotrophic lateral sclerosis. *J Biol Chem* 282**,** 23465-23472.

Feng, X., Peng, Y., Liu, M., and Cui, L. (2012). DL-3-n-butylphthalide extends survival by attenuating glial activation in a mouse model of amyotrophic lateral sclerosis. *Neuropharmacology* 62**,** 1004-1010.

Ferrante, R.J., Klein, A.M., Dedeoglu, A., and Beal, M.F. (2001). Therapeutic efficacy of EGb761 (Gingko biloba extract) in a transgenic mouse model of amyotrophic lateral sclerosis. *J Mol Neurosci* 17**,** 89-96.

Fontanilla, C.V., Wei, X., Zhao, L., Johnstone, B., Pascuzzi, R.M., Farlow, M.R., and Du, Y. (2012). Caffeic acid phenethyl ester extends survival of a mouse model of amyotrophic lateral sclerosis. *Neuroscience* 205**,** 185-193.

Fornai, F., Longone, P., Cafaro, L., Kastsiuchenka, O., Ferrucci, M., Manca, M.L., Lazzeri, G., Spalloni, A., Bellio, N., Lenzi, P., Modugno, N., Siciliano, G., Isidoro, C., Murri, L., Ruggieri, S., and Paparelli, A. (2008). Lithium delays progression of amyotrophic lateral sclerosis. *Proc Natl Acad Sci U S A* 105**,** 2052-2057.

Foust, K.D., Salazar, D.L., Likhite, S., Ferraiuolo, L., Ditsworth, D., Ilieva, H., Meyer, K., Schmelzer, L., Braun, L., Cleveland, D.W., and Kaspar, B.K. (2013). Therapeutic AAV9-mediated suppression of mutant SOD1 slows disease progression and extends survival in models of inherited ALS. *Mol Ther* 21**,** 2148-2159.

Garbuzova-Davis, S., Rodrigues, M.C., Mirtyl, S., Turner, S., Mitha, S., Sodhi, J., Suthakaran, S., Eve, D.J., Sanberg, C.D., Kuzmin-Nichols, N., and Sanberg, P.R. (2012). Multiple intravenous administrations of human umbilical cord blood cells benefit in a mouse model of ALS. *PLoS One* 7**,** e31254.

Garbuzova-Davis, S., Sanberg, C.D., Kuzmin-Nichols, N., Willing, A.E., Gemma, C., Bickford, P.C., Miller, C., Rossi, R., and Sanberg, P.R. (2008). Human umbilical cord blood treatment in a mouse model of ALS: optimization of cell dose. *PLoS One* 3**,** e2494.

Garbuzova-Davis, S., Willing, A.E., Saporta, S., Justen, E.B., Misiuta, I.E., Dellis, J., and Sanberg, P.R. (2006). Multiple transplants of hNT cells into the spinal cord of SOD1 mouse model of familial amyotrophic lateral sclerosis. *Amyotroph Lateral Scler* 7**,** 221-226.

Garbuzova-Davis, S., Willing, A.E., Zigova, T., Saporta, S., Justen, E.B., Lane, J.C., Hudson, J.E., Chen, N., Davis, C.D., and Sanberg, P.R. (2003). Intravenous administration of human umbilical cord blood cells in a mouse model of amyotrophic lateral sclerosis: distribution, migration, and differentiation. *J Hematother Stem Cell Res* 12**,** 255-270.

Gerber, Y.N., Privat, A., and Perrin, F.E. (2013). Gacyclidine improves the survival and reduces motor deficits in a mouse model of amyotrophic lateral sclerosis. *Front Cell Neurosci* 7**,** 280.

Gerber, Y.N., Sabourin, J.C., Hugnot, J.P., and Perrin, F.E. (2012). Unlike physical exercise, modified environment increases the lifespan of SOD1G93A mice however both conditions induce cellular changes. *PLoS One* 7**,** e45503.

Ghoddoussi, F., Galloway, M.P., Jambekar, A., Bame, M., Needleman, R., and Brusilow, W.S. (2010). Methionine sulfoximine, an inhibitor of glutamine synthetase, lowers brain glutamine and glutamate in a mouse model of ALS. *J Neurol Sci* 290**,** 41-47.

Gianforcaro, A., and Hamadeh, M.J. (2012). Dietary vitamin D3 supplementation at 10x the adequate intake improves functional capacity in the G93A transgenic mouse model of ALS, a pilot study. *CNS Neurosci Ther* 18**,** 547-557.

Gianforcaro, A., Solomon, J.A., and Hamadeh, M.J. (2013). Vitamin D(3) at 50x AI attenuates the decline in paw grip endurance, but not disease outcomes, in the G93A mouse model of ALS, and is toxic in females. *PLoS One* 8**,** e30243.

Gifondorwa, D.J., Jimenz-Moreno, R., Hayes, C.D., Rouhani, H., Robinson, M.B., Strupe, J.L., Caress, J., and Milligan, C. (2012). Administration of Recombinant Heat Shock Protein 70 Delays Peripheral Muscle Denervation in the SOD1(G93A) Mouse Model of Amyotrophic Lateral Sclerosis. *Neurol Res Int* 2012**,** 170426.

Gifondorwa, D.J., Robinson, M.B., Hayes, C.D., Taylor, A.R., Prevette, D.M., Oppenheim, R.W., Caress, J., and Milligan, C.E. (2007). Exogenous delivery of heat shock protein 70 increases lifespan in a mouse model of amyotrophic lateral sclerosis. *J Neurosci* 27**,** 13173-13180.

Glas, M., Popp, B., Angele, B., Koedel, U., Chahli, C., Schmalix, W.A., Anneser, J.M., Pfister, H.W., and Lorenzl, S. (2007). A role for the urokinase-type plasminogen activator system in amyotrophic lateral sclerosis. *Exp Neurol* 207**,** 350-356.

Gotkine, M., Rozenstein, L., Einstein, O., Abramsky, O., Argov, Z., and Rosenmann, H. (2013). Presymptomatic treatment with acetylcholinesterase antisense oligonucleotides prolongs survival in ALS (G93A-SOD1) mice. *Biomed Res Int* 2013**,** 845345.

Gould, T.W., Buss, R.R., Vinsant, S., Prevette, D., Sun, W., Knudson, C.M., Milligan, C.E., and Oppenheim, R.W. (2006). Complete dissociation of motor neuron death from motor dysfunction by Bax deletion in a mouse model of ALS. *J Neurosci* 26**,** 8774-8786.

Gowing, G., Dequen, F., Soucy, G., and Julien, J.P. (2006). Absence of tumor necrosis factor-alpha does not affect motor neuron disease caused by superoxide dismutase 1 mutations. *J Neurosci* 26**,** 11397-11402.

Grignaschi, G., Zennaro, E., Tortarolo, M., Calvaresi, N., and Bendotti, C. (2007). Erythropoietin does not preserve motor neurons in a mouse model of familial ALS. *Amyotroph Lateral Scler* 8**,** 31-35.

Groeneveld, G.J., De Leeuw Van Weenen, J., Van Muiswinkel, F.L., Veldman, H., Veldink, J.H., Wokke, J.H., Bar, P.R., and Van Den Berg, L.H. (2003). Zinc amplifies mSOD1-mediated toxicity in a transgenic mouse model of amyotrophic lateral sclerosis. *Neurosci Lett* 352**,** 175-178.

Groeneveld, G.J., Van Muiswinkel, F.L., De Leeuw Van Weenen, J., Blauw, H., Veldink, J.H., Wokke, J.H., Van Den Berg, L.H., and Bar, P.R. (2004). CGP 3466B has no effect on disease course of (G93A) mSOD1 transgenic mice. *Amyotroph Lateral Scler Other Motor Neuron Disord* 5**,** 220-225.

Gros-Louis, F., Soucy, G., Lariviere, R., and Julien, J.P. (2010). Intracerebroventricular infusion of monoclonal antibody or its derived Fab fragment against misfolded forms of SOD1 mutant delays mortality in a mouse model of ALS. *J Neurochem* 113**,** 1188-1199.

Guo, Y., Zhang, K., Wang, Q., Li, Z., Yin, Y., Xu, Q., Duan, W., and Li, C. (2011). Neuroprotective effects of diallyl trisulfide in SOD1-G93A transgenic mouse model of amyotrophic lateral sclerosis. *Brain Res* 1374**,** 110-115.

Gurney, M.E. (1997). Transgenic animal models of familial amyotrophic lateral sclerosis. *J Neurol* 244 Suppl 2**,** S15-20.

Gurney, M.E., Cutting, F.B., Zhai, P., Doble, A., Taylor, C.P., Andrus, P.K., and Hall, E.D. (1996). Benefit of vitamin E, riluzole, and gabapentin in a transgenic model of familial amyotrophic lateral sclerosis. *Ann Neurol* 39**,** 147-157.

Habisch, H.J., Janowski, M., Binder, D., Kuzma-Kozakiewicz, M., Widmann, A., Habich, A., Schwalenstocker, B., Hermann, A., Brenner, R., Lukomska, B., Domanska-Janik, K., Ludolph, A.C., and Storch, A. (2007a). Intrathecal application of neuroectodermally converted stem cells into a mouse model of ALS: limited intraparenchymal migration and survival narrows therapeutic effects. *J Neural Transm (Vienna)* 114**,** 1395-1406.

Habisch, H.J., Schwalenstocker, B., Danzeisen, R., Neuhaus, O., Hartung, H.P., and Ludolph, A. (2007b). Limited effects of glatiramer acetate in the high-copy number hSOD1-G93A mouse model of ALS. *Exp Neurol* 206**,** 288-295.

Hadano, S., Otomo, A., Kunita, R., Suzuki-Utsunomiya, K., Akatsuka, A., Koike, M., Aoki, M., Uchiyama, Y., Itoyama, Y., and Ikeda, J.E. (2010). Loss of ALS2/Alsin exacerbates motor dysfunction in a SOD1-expressing mouse ALS model by disturbing endolysosomal trafficking. *PLoS One* 5**,** e9805.

Hamadeh, M.J., Rodriguez, M.C., Kaczor, J.J., and Tarnopolsky, M.A. (2005). Caloric restriction transiently improves motor performance but hastens clinical onset of disease in the Cu/Zn-superoxide dismutase mutant G93A mouse. *Muscle Nerve* 31**,** 214-220.

Hamadeh, M.J., and Tarnopolsky, M.A. (2006). Transient caloric restriction in early adulthood hastens disease endpoint in male, but not female, Cu/Zn-SOD mutant G93A mice. *Muscle Nerve* 34**,** 709-719.

Han, S., Choi, J.R., Soon Shin, K., and Kang, S.J. (2012). Resveratrol upregulated heat shock proteins and extended the survival of G93A-SOD1 mice. *Brain Res* 1483**,** 112-117.

Holzbaur, E.L., Howland, D.S., Weber, N., Wallace, K., She, Y., Kwak, S., Tchistiakova, L.A., Murphy, E., Hinson, J., Karim, R., Tan, X.Y., Kelley, P., Mcgill, K.C., Williams, G., Hobbs, C., Doherty, P., Zaleska, M.M., Pangalos, M.N., and Walsh, F.S. (2006). Myostatin inhibition slows muscle atrophy in rodent models of amyotrophic lateral sclerosis. *Neurobiol Dis* 23**,** 697-707.

Hottinger, A.F., Fine, E.G., Gurney, M.E., Zurn, A.D., and Aebischer, P. (1997). The copper chelator d-penicillamine delays onset of disease and extends survival in a transgenic mouse model of familial amyotrophic lateral sclerosis. *Eur J Neurosci* 9**,** 1548-1551.

Jeon, G.S., Im, W., Shim, Y.M., Lee, M., Kim, M.J., Hong, Y.H., Seong, S.Y., Kim, M., and Sung, J.J. (2016). Neuroprotective Effect of Human Adipose Stem Cell-Derived Extract in Amyotrophic Lateral Sclerosis. *Neurochem Res* 41**,** 913-923.

Jiang, F., Desilva, S., and Turnbull, J. (2000). Beneficial effect of ginseng root in SOD-1 (G93A) transgenic mice. *J Neurol Sci* 180**,** 52-54.

Jiang, H.Q., Ren, M., Jiang, H.Z., Wang, J., Zhang, J., Yin, X., Wang, S.Y., Qi, Y., Wang, X.D., and Feng, H.L. (2014). Guanabenz delays the onset of disease symptoms, extends lifespan, improves motor performance and attenuates motor neuron loss in the SOD1 G93A mouse model of amyotrophic lateral sclerosis. *Neuroscience* 277**,** 132-138.

Joo, I.S., Hwang, D.H., Seok, J.I., Shin, S.K., and Kim, S.U. (2007). Oral administration of memantine prolongs survival in a transgenic mouse model of amyotrophic lateral sclerosis. *J Clin Neurol* 3**,** 181-186.

Kaneb, H.M., Sharp, P.S., Rahmani-Kondori, N., and Wells, D.J. (2011). Metformin treatment has no beneficial effect in a dose-response survival study in the SOD1(G93A) mouse model of ALS and is harmful in female mice. *PLoS One* 6**,** e24189.

Kaspar, B.K., Frost, L.M., Christian, L., Umapathi, P., and Gage, F.H. (2005). Synergy of insulin-like growth factor-1 and exercise in amyotrophic lateral sclerosis. *Ann Neurol* 57**,** 649-655.

Katsumata, R., Ishigaki, S., Katsuno, M., Kawai, K., Sone, J., Huang, Z., Adachi, H., Tanaka, F., Urano, F., and Sobue, G. (2012). c-Abl inhibition delays motor neuron degeneration in the G93A mouse, an animal model of amyotrophic lateral sclerosis. *PLoS One* 7**,** e46185.

Keller, A.F., Gravel, M., and Kriz, J. (2011). Treatment with minocycline after disease onset alters astrocyte reactivity and increases microgliosis in SOD1 mutant mice. *Exp Neurol* 228**,** 69-79.

Kelly, J.A., Boyle, N.T., Cole, N., Slator, G.R., Colivicchi, M.A., Stefanini, C., Gobbo, O.L., Scalabrino, G.A., Ryan, S.M., Elamin, M., Walsh, C., Vajda, A., Goggin, M.M., Campbell, M., Mash, D.C., O'mara, S.M., Brayden, D.J., Callanan, J.J., Tipton, K.F., Della Corte, L., Hunter, J., O'boyle, K.M., Williams, C.H., and Hardiman, O. (2015). First-in-class thyrotropin-releasing hormone (TRH)-based compound binds to a pharmacologically distinct TRH receptor subtype in human brain and is effective in neurodegenerative models. *Neuropharmacology* 89**,** 193-203.

Kiaei, M., Kipiani, K., Chen, J., Calingasan, N.Y., and Beal, M.F. (2005a). Peroxisome proliferator-activated receptor-gamma agonist extends survival in transgenic mouse model of amyotrophic lateral sclerosis. *Exp Neurol* 191**,** 331-336.

Kiaei, M., Kipiani, K., Petri, S., Chen, J., Calingasan, N.Y., and Beal, M.F. (2005b). Celastrol blocks neuronal cell death and extends life in transgenic mouse model of amyotrophic lateral sclerosis. *Neurodegener Dis* 2**,** 246-254.

Kiaei, M., Kipiani, K., Petri, S., Choi, D.K., Chen, J., Calingasan, N.Y., and Beal, M.F. (2005c). Integrative role of cPLA with COX-2 and the effect of non-steriodal anti-inflammatory drugs in a transgenic mouse model of amyotrophic lateral sclerosis. *J Neurochem* 93**,** 403-411.

Kiaei, M., Petri, S., Kipiani, K., Gardian, G., Choi, D.K., Chen, J., Calingasan, N.Y., Schafer, P., Muller, G.W., Stewart, C., Hensley, K., and Beal, M.F. (2006). Thalidomide and lenalidomide extend survival in a transgenic mouse model of amyotrophic lateral sclerosis. *J Neurosci* 26**,** 2467-2473.

Kieran, D., Kalmar, B., Dick, J.R., Riddoch-Contreras, J., Burnstock, G., and Greensmith, L. (2004). Treatment with arimoclomol, a coinducer of heat shock proteins, delays disease progression in ALS mice. *Nat Med* 10**,** 402-405.

Kieran, D., Sebastia, J., Greenway, M.J., King, M.A., Connaughton, D., Concannon, C.G., Fenner, B., Hardiman, O., and Prehn, J.H. (2008). Control of motoneuron survival by angiogenin. *J Neurosci* 28**,** 14056-14061.

Kim, H., Kim, H.Y., Choi, M.R., Hwang, S., Nam, K.H., Kim, H.C., Han, J.S., Kim, K.S., Yoon, H.S., and Kim, S.H. (2010). Dose-dependent efficacy of ALS-human mesenchymal stem cells transplantation into cisterna magna in SOD1-G93A ALS mice. *Neurosci Lett* 468**,** 190-194.

Kim, J., Kim, T.Y., Cho, K.S., Kim, H.N., and Koh, J.Y. (2013). Autophagy activation and neuroprotection by progesterone in the G93A-SOD1 transgenic mouse model of amyotrophic lateral sclerosis. *Neurobiol Dis* 59**,** 80-85.

Kim, J., Kim, T.Y., Hwang, J.J., Lee, J.Y., Shin, J.H., Gwag, B.J., and Koh, J.Y. (2009). Accumulation of labile zinc in neurons and astrocytes in the spinal cords of G93A SOD-1 transgenic mice. *Neurobiol Dis* 34**,** 221-229.

Kirkinezos, I.G., Hernandez, D., Bradley, W.G., and Moraes, C.T. (2003). Regular exercise is beneficial to a mouse model of amyotrophic lateral sclerosis. *Ann Neurol* 53**,** 804-807.

Klivenyi, P., Ferrante, R.J., Matthews, R.T., Bogdanov, M.B., Klein, A.M., Andreassen, O.A., Mueller, G., Wermer, M., Kaddurah-Daouk, R., and Beal, M.F. (1999). Neuroprotective effects of creatine in a transgenic animal model of amyotrophic lateral sclerosis. *Nat Med* 5**,** 347-350.

Klivenyi, P., Kiaei, M., Gardian, G., Calingasan, N.Y., and Beal, M.F. (2004). Additive neuroprotective effects of creatine and cyclooxygenase 2 inhibitors in a transgenic mouse model of amyotrophic lateral sclerosis. *J Neurochem* 88**,** 576-582.

Knippenberg, S., Skripuletz, T., Rath, K.J., Thau, N., Gudi, V., Pul, R., Korner, S., Dengler, R., Stangel, M., and Petri, S. (2013). CDP-choline is not protective in the SOD1-G93A mouse model of ALS. *Amyotroph Lateral Scler Frontotemporal Degener* 14**,** 284-290.

Knippenberg, S., Thau, N., Dengler, R., Brinker, T., and Petri, S. (2012a). Intracerebroventricular injection of encapsulated human mesenchymal cells producing glucagon-like peptide 1 prolongs survival in a mouse model of ALS. *PLoS One* 7**,** e36857.

Knippenberg, S., Thau, N., Schwabe, K., Dengler, R., Schambach, A., Hass, R., and Petri, S. (2012b). Intraspinal injection of human umbilical cord blood-derived cells is neuroprotective in a transgenic mouse model of amyotrophic lateral sclerosis. *Neurodegener Dis* 9**,** 107-120.

Koh, S.H., Kim, Y., Kim, H.Y., Cho, G.W., Kim, K.S., and Kim, S.H. (2007a). Recombinant human erythropoietin suppresses symptom onset and progression of G93A-SOD1 mouse model of ALS by preventing motor neuron death and inflammation. *Eur J Neurosci* 25**,** 1923-1930.

Koh, S.H., Kim, Y., Kim, H.Y., Hwang, S., Lee, C.H., and Kim, S.H. (2007b). Inhibition of glycogen synthase kinase-3 suppresses the onset of symptoms and disease progression of G93A-SOD1 mouse model of ALS. *Exp Neurol* 205**,** 336-346.

Koh, S.H., Lee, S.M., Kim, H.Y., Lee, K.Y., Lee, Y.J., Kim, H.T., Kim, J., Kim, M.H., Hwang, M.S., Song, C., Yang, K.W., Lee, K.W., Kim, S.H., and Kim, O.H. (2006). The effect of epigallocatechin gallate on suppressing disease progression of ALS model mice. *Neurosci Lett* 395**,** 103-107.

Kong, Q., Carothers, S., Chang, Y., and Glenn Lin, C.L. (2012). The importance of preclinical trial timing - a potential reason for the disconnect between mouse studies and human clinical trials in ALS. *CNS Neurosci Ther* 18**,** 791-793.

Kupershmidt, L., Weinreb, O., Amit, T., Mandel, S., Carri, M.T., and Youdim, M.B. (2009). Neuroprotective and neuritogenic activities of novel multimodal iron-chelating drugs in motor-neuron-like NSC-34 cells and transgenic mouse model of amyotrophic lateral sclerosis. *FASEB J* 23**,** 3766-3779.

Kust, B.M., Brouwer, N., Mantingh, I.J., Boddeke, H.W., and Copray, J.C. (2003). Reduced p75NTR expression delays disease onset only in female mice of a transgenic model of familial amyotrophic lateral sclerosis. *Amyotroph Lateral Scler Other Motor Neuron Disord* 4**,** 100-105.

Lee, J., Ryu, H., and Kowall, N.W. (2009). Motor neuronal protection by L-arginine prolongs survival of mutant SOD1 (G93A) ALS mice. *Biochem Biophys Res Commun* 384**,** 524-529.

Lee, J.C., Seong, J., Kim, S.H., Lee, S.J., Cho, Y.J., An, J., Nam, D.H., Joo, K.M., and Cha, C.I. (2012). Replacement of microglial cells using Clodronate liposome and bone marrow transplantation in the central nervous system of SOD1(G93A) transgenic mice as an in vivo model of amyotrophic lateral sclerosis. *Biochem Biophys Res Commun* 418**,** 359-365.

Lee, J.K., Shin, J.H., Gwag, B.J., and Choi, E.J. (2015). Iron accumulation promotes TACE-mediated TNF-alpha secretion and neurodegeneration in a mouse model of ALS. *Neurobiol Dis* 80**,** 63-69.

Li, J., Paulson, J.M., Ye, F.D., Sung, M., Hollenberg, A.N., and Rutkove, S.B. (2012a). Reducing systemic hypermetabolism by inducing hypothyroidism does not prolong survival in the SOD1-G93A mouse. *Amyotroph Lateral Scler* 13**,** 372-377.

Li, M., Ona, V.O., Guegan, C., Chen, M., Jackson-Lewis, V., Andrews, L.J., Olszewski, A.J., Stieg, P.E., Lee, J.P., Przedborski, S., and Friedlander, R.M. (2000). Functional role of caspase-1 and caspase-3 in an ALS transgenic mouse model. *Science* 288**,** 335-339.

Li, S., Sheng, J., Hu, J.K., Yu, W., Kishikawa, H., Hu, M.G., Shima, K., Wu, D., Xu, Z., Xin, W., Sims, K.B., Landers, J.E., Brown, R.H., Jr., and Hu, G.F. (2013). Ribonuclease 4 protects neuron degeneration by promoting angiogenesis, neurogenesis, and neuronal survival under stress. *Angiogenesis* 16**,** 387-404.

Li, Y., Chigurupati, S., Holloway, H.W., Mughal, M., Tweedie, D., Bruestle, D.A., Mattson, M.P., Wang, Y., Harvey, B.K., Ray, B., Lahiri, D.K., and Greig, N.H. (2012b). Exendin-4 ameliorates motor neuron degeneration in cellular and animal models of amyotrophic lateral sclerosis. *PLoS One* 7**,** e32008.

Li, Y., Guo, Y., Wang, X., Yu, X., Duan, W., Hong, K., Wang, J., Han, H., and Li, C. (2015). Trehalose decreases mutant SOD1 expression and alleviates motor deficiency in early but not end-stage amyotrophic lateral sclerosis in a SOD1-G93A mouse model. *Neuroscience* 298**,** 12-25.

Liebetanz, D., Hagemann, K., Von Lewinski, F., Kahler, E., and Paulus, W. (2004). Extensive exercise is not harmful in amyotrophic lateral sclerosis. *Eur J Neurosci* 20**,** 3115-3120.

Lincecum, J.M., Vieira, F.G., Wang, M.Z., Thompson, K., De Zutter, G.S., Kidd, J., Moreno, A., Sanchez, R., Carrion, I.J., Levine, B.A., Al-Nakhala, B.M., Sullivan, S.M., Gill, A., and Perrin, S. (2010). From transcriptome analysis to therapeutic anti-CD40L treatment in the SOD1 model of amyotrophic lateral sclerosis. *Nat Genet* 42**,** 392-399.

Locatelli, F., Corti, S., Papadimitriou, D., Fortunato, F., Del Bo, R., Donadoni, C., Nizzardo, M., Nardini, M., Salani, S., Ghezzi, S., Strazzer, S., Bresolin, N., and Comi, G.P. (2007). Fas small interfering RNA reduces motoneuron death in amyotrophic lateral sclerosis mice. *Ann Neurol* 62**,** 81-92.

Lorenzl, S., Narr, S., Angele, B., Krell, H.W., Gregorio, J., Kiaei, M., Pfister, H.W., and Beal, M.F. (2006). The matrix metalloproteinases inhibitor Ro 28-2653 [correction of Ro 26-2853] extends survival in transgenic ALS mice. *Exp Neurol* 200**,** 166-171.

Mahoney, D.J., Rodriguez, C., Devries, M., Yasuda, N., and Tarnopolsky, M.A. (2004). Effects of high-intensity endurance exercise training in the G93A mouse model of amyotrophic lateral sclerosis. *Muscle Nerve* 29**,** 656-662.

Mancuso, R., Del Valle, J., Modol, L., Martinez, A., Granado-Serrano, A.B., Ramirez-Nunez, O., Pallas, M., Portero-Otin, M., Osta, R., and Navarro, X. (2014a). Resveratrol improves motoneuron function and extends survival in SOD1(G93A) ALS mice. *Neurotherapeutics* 11**,** 419-432.

Mancuso, R., Del Valle, J., Morell, M., Pallas, M., Osta, R., and Navarro, X. (2014b). Lack of synergistic effect of resveratrol and sigma-1 receptor agonist (PRE-084) in SOD1G(9)(3)A ALS mice: overlapping effects or limited therapeutic opportunity? *Orphanet J Rare Dis* 9**,** 78.

Mancuso, R., Olivan, S., Rando, A., Casas, C., Osta, R., and Navarro, X. (2012). Sigma-1R agonist improves motor function and motoneuron survival in ALS mice. *Neurotherapeutics* 9**,** 814-826.

Markert, C.D., Kim, E., Gifondorwa, D.J., Childers, M.K., and Milligan, C.E. (2010). A single-dose resveratrol treatment in a mouse model of amyotrophic lateral sclerosis. *J Med Food* 13**,** 1081-1085.

Martin, L.J., and Liu, Z. (2007). Adult olfactory bulb neural precursor cell grafts provide temporary protection from motor neuron degeneration, improve motor function, and extend survival in amyotrophic lateral sclerosis mice. *J Neuropathol Exp Neurol* 66**,** 1002-1018.

Martin, L.J., Liu, Z., Chen, K., Price, A.C., Pan, Y., Swaby, J.A., and Golden, W.C. (2007). Motor neuron degeneration in amyotrophic lateral sclerosis mutant superoxide dismutase-1 transgenic mice: mechanisms of mitochondriopathy and cell death. *J Comp Neurol* 500**,** 20-46.

Martinez, J.A., Francis, G.J., Liu, W.Q., Pradzinsky, N., Fine, J., Wilson, M., Hanson, L.R., Frey, W.H., 2nd, Zochodne, D., Gordon, T., and Toth, C. (2008). Intranasal delivery of insulin and a nitric oxide synthase inhibitor in an experimental model of amyotrophic lateral sclerosis. *Neuroscience* 157**,** 908-925.

Matthews, R.T., Yang, L., Browne, S., Baik, M., and Beal, M.F. (1998). Coenzyme Q10 administration increases brain mitochondrial concentrations and exerts neuroprotective effects. *Proc Natl Acad Sci U S A* 95**,** 8892-8897.

Mattson, M.P., Cutler, R.G., and Camandola, S. (2007). Energy intake and amyotrophic lateral sclerosis. *Neuromolecular Med* 9**,** 17-20.

Mccrate, M.E., and Kaspar, B.K. (2008). Physical activity and neuroprotection in amyotrophic lateral sclerosis. *Neuromolecular Med* 10**,** 108-117.

Mead, R.J., Higginbottom, A., Allen, S.P., Kirby, J., Bennett, E., Barber, S.C., Heath, P.R., Coluccia, A., Patel, N., Gardner, I., Brancale, A., Grierson, A.J., and Shaw, P.J. (2013). S[+] Apomorphine is a CNS penetrating activator of the Nrf2-ARE pathway with activity in mouse

and patient fibroblast models of amyotrophic lateral sclerosis. *Free Radic Biol Med* 61, 438-452.

Messi, M.L., Clark, H.M., Prevette, D.M., Oppenheim, R.W., and Delbono, O. (2007). The lack of effect of specific overexpression of IGF-1 in the central nervous system or skeletal muscle on pathophysiology in the G93A SOD-1 mouse model of ALS. *Exp Neurol* 207, 52-63.

Miller, T.M., Kim, S.H., Yamanaka, K., Hester, M., Umapathi, P., Arnson, H., Rizo, L., Mendell, J.R., Gage, F.H., Cleveland, D.W., and Kaspar, B.K. (2006). Gene transfer demonstrates that muscle is not a primary target for non-cell-autonomous toxicity in familial amyotrophic lateral sclerosis. *Proc Natl Acad Sci U S A* 103, 19546-19551.

Miquel, E., Cassina, A., Martinez-Palma, L., Bolatto, C., Trias, E., Gandelman, M., Radi, R., Barbeito, L., and Cassina, P. (2012). Modulation of astrocytic mitochondrial function by dichloroacetate improves survival and motor performance in inherited amyotrophic lateral sclerosis. *PLoS One* 7, e34776.

Miquel, E., Cassina, A., Martinez-Palma, L., Souza, J.M., Bolatto, C., Rodriguez-Bottero, S., Logan, A., Smith, R.A., Murphy, M.P., Barbeito, L., Radi, R., and Cassina, P. (2014). Neuroprotective effects of the mitochondria-targeted antioxidant MitoQ in a model of inherited amyotrophic lateral sclerosis. *Free Radic Biol Med* 70, 204-213.

Moges, H., Vasconcelos, O.M., Campbell, W.W., Borke, R.C., Mccoy, J.A., Kaczmarczyk, L., Feng, J., and Anders, J.J. (2009). Light therapy and supplementary Riboflavin in the SOD1 transgenic mouse model of familial amyotrophic lateral sclerosis (FALS). *Lasers Surg Med* 41, 52-59.

Mohajeri, M.H., Figlewicz, D.A., and Bohn, M.C. (1999). Intramuscular grafts of myoblasts genetically modified to secrete glial cell line-derived neurotrophic factor prevent motoneuron loss and disease progression in a mouse model of familial amyotrophic lateral sclerosis. *Hum Gene Ther* 10, 1853-1866.

Moreno-Igoa, M., Calvo, A.C., Ciriza, J., Munoz, M.J., Zaragoza, P., and Osta, R. (2012). Non-viral gene delivery of the GDNF, either alone or fused to the C-fragment of tetanus toxin protein, prolongs survival in a mouse ALS model. *Restor Neurol Neurosci* 30, 69-80.

Moreno-Igoa, M., Calvo, A.C., Penas, C., Manzano, R., Olivan, S., Munoz, M.J., Mancuso, R., Zaragoza, P., Aguilera, J., Navarro, X., and Osta Pinzolas, R. (2010). Fragment C of tetanus toxin, more than a carrier. Novel perspectives in non-viral ALS gene therapy. *J Mol Med (Berl)* 88, 297-308.

Morrison, B.M., Lachey, J.L., Warsing, L.C., Ting, B.L., Pullen, A.E., Underwood, K.W., Kumar, R., Sako, D., Grinberg, A., Wong, V., Colantuoni, E., Seehra, J.S., and Wagner, K.R. (2009). A soluble activin type IIB receptor improves function in a mouse model of amyotrophic lateral sclerosis. *Exp Neurol* 217, 258-268.

Nagahara, Y., Shimazawa, M., Tanaka, H., Ono, Y., Noda, Y., Ohuchi, K., Tsuruma, K., Katsuno, M., Sobue, G., and Hara, H. (2015). Glycoprotein nonmetastatic melanoma protein B ameliorates skeletal muscle lesions in a SOD1G93A mouse model of amyotrophic lateral sclerosis. *J Neurosci Res* 93, 1552-1566.

Neymotin, A., Petri, S., Calingasan, N.Y., Wille, E., Schafer, P., Stewart, C., Hensley, K., Beal, M.F., and Kiaei, M. (2009). Lenalidomide (Revlimid) administration at symptom onset is neuroprotective in a mouse model of amyotrophic lateral sclerosis. *Exp Neurol* 220, 191-197.

Ng, S.K., Higashimori, H., Tolman, M., and Yang, Y. (2015). Suppression of adenosine 2a receptor (A2aR)-mediated adenosine signaling improves disease phenotypes in a mouse model of amyotrophic lateral sclerosis. *Exp Neurol* 267, 115-122.

Ohta, Y., Kamiya, T., Nagai, M., Nagata, T., Morimoto, N., Miyazaki, K., Murakami, T., Kurata, T., Takehisa, Y., Ikeda, Y., Asoh, S., Ohta, S., and Abe, K. (2008). Therapeutic benefits of

intrathecal protein therapy in a mouse model of amyotrophic lateral sclerosis. *J Neurosci Res* 86, 3028-3037.
Olsen, M.K., Roberds, S.L., Ellerbrock, B.R., Fleck, T.J., Mckinley, D.K., and Gurney, M.E. (2001). Disease mechanisms revealed by transcription profiling in SOD1-G93A transgenic mouse spinal cord. *Ann Neurol* 50, 730-740.
Ono, Y., Tanaka, H., Takata, M., Nagahara, Y., Noda, Y., Tsuruma, K., Shimazawa, M., Hozumi, I., and Hara, H. (2014). SA4503, a sigma-1 receptor agonist, suppresses motor neuron damage in in vitro and in vivo amyotrophic lateral sclerosis models. *Neurosci Lett* 559, 174-178.
Pamphlett, R., Todd, E., Vink, R., Mcquilty, R., and Cheema, S.S. (2003). Magnesium supplementation does not delay disease onset or increase survival in a mouse model of familial ALS. *J Neurol Sci* 216, 95-98.
Patel, B.P., Safdar, A., Raha, S., Tarnopolsky, M.A., and Hamadeh, M.J. (2010). Caloric restriction shortens lifespan through an increase in lipid peroxidation, inflammation and apoptosis in the G93A mouse, an animal model of ALS. *PLoS One* 5, e9386.
Perez-Garcia, M.J., and Burden, S.J. (2012). Increasing MuSK activity delays denervation and improves motor function in ALS mice. *Cell Rep* 2, 497-502.
Petri, S., Calingasan, N.Y., Alsaied, O.A., Wille, E., Kiaei, M., Friedman, J.E., Baranova, O., Chavez, J.C., and Beal, M.F. (2007). The lipophilic metal chelators DP-109 and DP-460 are neuroprotective in a transgenic mouse model of amyotrophic lateral sclerosis. *J Neurochem* 102, 991-1000.
Petri, S., Kiaei, M., Damiano, M., Hiller, A., Wille, E., Manfredi, G., Calingasan, N.Y., Szeto, H.H., and Beal, M.F. (2006a). Cell-permeable peptide antioxidants as a novel therapeutic approach in a mouse model of amyotrophic lateral sclerosis. *J Neurochem* 98, 1141-1148.
Petri, S., Kiaei, M., Kipiani, K., Chen, J., Calingasan, N.Y., Crow, J.P., and Beal, M.F. (2006b). Additive neuroprotective effects of a histone deacetylase inhibitor and a catalytic antioxidant in a transgenic mouse model of amyotrophic lateral sclerosis. *Neurobiol Dis* 22, 40-49.
Pieper, A.A., Mcknight, S.L., and Ready, J.M. (2014). P7C3 and an unbiased approach to drug discovery for neurodegenerative diseases. *Chem Soc Rev* 43, 6716-6726.
Pompl, P.N., Ho, L., Bianchi, M., Mcmanus, T., Qin, W., and Pasinetti, G.M. (2003). A therapeutic role for cyclooxygenase-2 inhibitors in a transgenic mouse model of amyotrophic lateral sclerosis. *FASEB J* 17, 725-727.
Potenza, R.L., Armida, M., Ferrante, A., Pezzola, A., Matteucci, A., Puopolo, M., and Popoli, P. (2013). Effects of chronic caffeine intake in a mouse model of amyotrophic lateral sclerosis. *J Neurosci Res* 91, 585-592.
Poulletier De Gannes, F., Ruffie, G., Taxile, M., Ladeveze, E., Hurtier, A., Haro, E., Duleu, S., Charlet De Sauvage, R., Billaudel, B., Geffard, M., Veyret, B., and Lagroye, I. (2009). Amyotrophic lateral sclerosis (ALS) and extremely-low frequency (ELF) magnetic fields: a study in the SOD-1 transgenic mouse model. *Amyotroph Lateral Scler* 10, 370-373.
Rabinovich-Toidman, P., Becker, M., Barbiro, B., and Solomon, B. (2012). Inhibition of amyloid precursor protein beta-secretase cleavage site affects survival and motor functions of amyotrophic lateral sclerosis transgenic mice. *Neurodegener Dis* 10, 30-33.
Rabinovich-Toidman, P., Rabinovich-Nikitin, I., Ezra, A., Barbiro, B., Fogel, H., Slutsky, I., and Solomon, B. (2015). Mutant SOD1 Increases APP Expression and Phosphorylation in Cellular and Animal Models of ALS. *PLoS One* 10, e0143420.
Raman, C., Mcallister, S.D., Rizvi, G., Patel, S.G., Moore, D.H., and Abood, M.E. (2004). Amyotrophic lateral sclerosis: delayed disease progression in mice by treatment with a cannabinoid. *Amyotroph Lateral Scler Other Motor Neuron Disord* 5, 33-39.

Ryu, H., Smith, K., Camelo, S.I., Carreras, I., Lee, J., Iglesias, A.H., Dangond, F., Cormier, K.A., Cudkowicz, M.E., Brown, R.H., Jr., and Ferrante, R.J. (2005). Sodium phenylbutyrate prolongs survival and regulates expression of anti-apoptotic genes in transgenic amyotrophic lateral sclerosis mice. *J Neurochem* 93**,** 1087-1098.

Saenger, S., Holtmann, B., Nilges, M.R., Schroeder, S., Hoeflich, A., Kletzl, H., Spooren, W., Ostrowitzki, S., Hanania, T., Sendtner, M., and Metzger, F. (2012). Functional improvement in mouse models of familial amyotrophic lateral sclerosis by PEGylated insulin-like growth factor I treatment depends on disease severity. *Amyotroph Lateral Scler* 13**,** 418-429.

Schutz, B., Reimann, J., Dumitrescu-Ozimek, L., Kappes-Horn, K., Landreth, G.E., Schurmann, B., Zimmer, A., and Heneka, M.T. (2005). The oral antidiabetic pioglitazone protects from neurodegeneration and amyotrophic lateral sclerosis-like symptoms in superoxide dismutase-G93A transgenic mice. *J Neurosci* 25**,** 7805-7812.

Sekiya, M., Ichiyanagi, T., Ikeshiro, Y., and Yokozawa, T. (2009). The Chinese prescription Wen-Pi-Tang extract delays disease onset in amyotrophic lateral sclerosis model mice while attenuating the activation of glial cells in the spinal cord. *Biol Pharm Bull* 32**,** 382-388.

Sharp, P.S., Akbar, M.T., Bouri, S., Senda, A., Joshi, K., Chen, H.J., Latchman, D.S., Wells, D.J., and De Belleroche, J. (2008). Protective effects of heat shock protein 27 in a model of ALS occur in the early stages of disease progression. *Neurobiol Dis* 30**,** 42-55.

Shimazawa, M., Tanaka, H., Ito, Y., Morimoto, N., Tsuruma, K., Kadokura, M., Tamura, S., Inoue, T., Yamada, M., Takahashi, H., Warita, H., Aoki, M., and Hara, H. (2010). An inducer of VGF protects cells against ER stress-induced cell death and prolongs survival in the mutant SOD1 animal models of familial ALS. *PLoS One* 5**,** e15307.

Shin, J.H., Cho, S.I., Lim, H.R., Lee, J.K., Lee, Y.A., Noh, J.S., Joo, I.S., Kim, K.W., and Gwag, B.J. (2007). Concurrent administration of Neu2000 and lithium produces marked improvement of motor neuron survival, motor function, and mortality in a mouse model of amyotrophic lateral sclerosis. *Mol Pharmacol* 71**,** 965-975.

Shin, J.H., Lee, Y.A., Lee, J.K., Lee, Y.B., Cho, W., Im, D.S., Lee, J.H., Yun, B.S., Springer, J.E., and Gwag, B.J. (2012). Concurrent blockade of free radical and microsomal prostaglandin E synthase-1-mediated PGE2 production improves safety and efficacy in a mouse model of amyotrophic lateral sclerosis. *J Neurochem* 122**,** 952-961.

Solomon, J.N., Lewis, C.A., Ajami, B., Corbel, S.Y., Rossi, F.M., and Krieger, C. (2006). Origin and distribution of bone marrow-derived cells in the central nervous system in a mouse model of amyotrophic lateral sclerosis. *Glia* 53**,** 744-753.

Song, L., Chen, L., Zhang, X., Li, J., and Le, W. (2014). Resveratrol ameliorates motor neuron degeneration and improves survival in SOD1(G93A) mouse model of amyotrophic lateral sclerosis. *Biomed Res Int* 2014**,** 483501.

Stam, N.C., Nithianantharajah, J., Howard, M.L., Atkin, J.D., Cheema, S.S., and Hannan, A.J. (2008). Sex-specific behavioural effects of environmental enrichment in a transgenic mouse model of amyotrophic lateral sclerosis. *Eur J Neurosci* 28**,** 717-723.

Tada, S., Okuno, T., Yasui, T., Nakatsuji, Y., Sugimoto, T., Kikutani, H., and Sakoda, S. (2011). Deleterious effects of lymphocytes at the early stage of neurodegeneration in an animal model of amyotrophic lateral sclerosis. *J Neuroinflammation* 8**,** 19.

Takata, M., Tanaka, H., Kimura, M., Nagahara, Y., Tanaka, K., Kawasaki, K., Seto, M., Tsuruma, K., Shimazawa, M., and Hara, H. (2013). Fasudil, a rho kinase inhibitor, limits motor neuron loss in experimental models of amyotrophic lateral sclerosis. *Br J Pharmacol* 170**,** 341-351.

Tokuda, E., Okawa, E., Watanabe, S., Ono, S., and Marklund, S.L. (2013). Dysregulation of intracellular copper homeostasis is common to transgenic mice expressing human mutant

superoxide dismutase-1s regardless of their copper-binding abilities. *Neurobiol Dis* 54, 308-319.

Tokuda, E., Ono, S., Ishige, K., Watanabe, S., Okawa, E., Ito, Y., and Suzuki, T. (2008). Ammonium tetrathiomolybdate delays onset, prolongs survival, and slows progression of disease in a mouse model for amyotrophic lateral sclerosis. *Exp Neurol* 213, 122-128.

Tonges, L., Gunther, R., Suhr, M., Jansen, J., Balck, A., Saal, K.A., Barski, E., Nientied, T., Gotz, A.A., Koch, J.C., Mueller, B.K., Weishaupt, J.H., Sereda, M.W., Hanisch, U.K., Bahr, M., and Lingor, P. (2014). Rho kinase inhibition modulates microglia activation and improves survival in a model of amyotrophic lateral sclerosis. *Glia* 62, 217-232.

Towne, C., Setola, V., Schneider, B.L., and Aebischer, P. (2011). Neuroprotection by gene therapy targeting mutant SOD1 in individual pools of motor neurons does not translate into therapeutic benefit in fALS mice. *Mol Ther* 19, 274-283.

Trieu, V.N., Liu, R., Liu, X.P., and Uckun, F.M. (2000). A specific inhibitor of janus kinase-3 increases survival in a transgenic mouse model of amyotrophic lateral sclerosis. *Biochem Biophys Res Commun* 267, 22-25.

Trumbull, K.A., Mcallister, D., Gandelman, M.M., Fung, W.Y., Lew, T., Brennan, L., Lopez, N., Morre, J., Kalyanaraman, B., and Beckman, J.S. (2012). Diapocynin and apocynin administration fails to significantly extend survival in G93A SOD1 ALS mice. *Neurobiol Dis* 45, 137-144.

Turner, B.J., Ackerley, S., Davies, K.E., and Talbot, K. (2010). Dismutase-competent SOD1 mutant accumulation in myelinating Schwann cells is not detrimental to normal or transgenic ALS model mice. *Hum Mol Genet* 19, 815-824.

Turner, B.J., Lopes, E.C., and Cheema, S.S. (2003). The serotonin precursor 5-hydroxytryptophan delays neuromuscular disease in murine familial amyotrophic lateral sclerosis. *Amyotroph Lateral Scler Other Motor Neuron Disord* 4, 171-176.

Uccelli, A., Milanese, M., Principato, M.C., Morando, S., Bonifacino, T., Vergani, L., Giunti, D., Voci, A., Carminati, E., Giribaldi, F., Caponnetto, C., and Bonanno, G. (2012). Intravenous mesenchymal stem cells improve survival and motor function in experimental amyotrophic lateral sclerosis. *Mol Med* 18, 794-804.

Vaknin, I., Kunis, G., Miller, O., Butovsky, O., Bukshpan, S., Beers, D.R., Henkel, J.S., Yoles, E., Appel, S.H., and Schwartz, M. (2011). Excess circulating alternatively activated myeloid (M2) cells accelerate ALS progression while inhibiting experimental autoimmune encephalomyelitis. *PLoS One* 6, e26921.

Van Damme, P., Leyssen, M., Callewaert, G., Robberecht, W., and Van Den Bosch, L. (2003). The AMPA receptor antagonist NBQX prolongs survival in a transgenic mouse model of amyotrophic lateral sclerosis. *Neurosci Lett* 343, 81-84.

Van Den Bosch, L., Tilkin, P., Lemmens, G., and Robberecht, W. (2002). Minocycline delays disease onset and mortality in a transgenic model of ALS. *Neuroreport* 13, 1067-1070.

Veldink, J.H., Bar, P.R., Joosten, E.A., Otten, M., Wokke, J.H., and Van Den Berg, L.H. (2003). Sexual differences in onset of disease and response to exercise in a transgenic model of ALS. *Neuromuscul Disord* 13, 737-743.

Venerosi, A., Martire, A., Rungi, A., Pieri, M., Ferrante, A., Zona, C., Popoli, P., and Calamandrei, G. (2011). Complex behavioral and synaptic effects of dietary branched chain amino acids in a mouse model of amyotrophic lateral sclerosis. *Mol Nutr Food Res* 55, 541-552.

Venkova, K., Christov, A., Kamaluddin, Z., Kobalka, P., Siddiqui, S., and Hensley, K. (2014). Semaphorin 3A signaling through neuropilin-1 is an early trigger for distal axonopathy in the SOD1G93A mouse model of amyotrophic lateral sclerosis. *J Neuropathol Exp Neurol* 73, 702-713.

Vieira, F.G., Ping, Q., Moreno, A.J., Kidd, J.D., Thompson, K., Jiang, B., Lincecum, J.M., Wang, M.Z., De Zutter, G.S., Tassinari, V.R., Levine, B., Hatzipetros, T., Gill, A., and Perrin, S. (2015). Guanabenz Treatment Accelerates Disease in a Mutant SOD1 Mouse Model of ALS. *PLoS One* 10, e0135570.

Wang, H., Guan, Y., Wang, X., Smith, K., Cormier, K., Zhu, S., Stavrovskaya, I.G., Huo, C., Ferrante, R.J., Kristal, B.S., and Friedlander, R.M. (2007a). Nortriptyline delays disease onset in models of chronic neurodegeneration. *Eur J Neurosci* 26, 633-641.

Wang, L.J., Lu, Y.Y., Muramatsu, S., Ikeguchi, K., Fujimoto, K., Okada, T., Mizukami, H., Matsushita, T., Hanazono, Y., Kume, A., Nagatsu, T., Ozawa, K., and Nakano, I. (2002). Neuroprotective effects of glial cell line-derived neurotrophic factor mediated by an adeno-associated virus vector in a transgenic animal model of amyotrophic lateral sclerosis. *J Neurosci* 22, 6920-6928.

Wang, Q., Zhang, X., Chen, S., Zhang, X., Zhang, S., Youdium, M., and Le, W. (2011). Prevention of motor neuron degeneration by novel iron chelators in SOD1(G93A) transgenic mice of amyotrophic lateral sclerosis. *Neurodegener Dis* 8, 310-321.

Wang, Y., Mao, X.O., Xie, L., Banwait, S., Marti, H.H., Greenberg, D.A., and Jin, K. (2007b). Vascular endothelial growth factor overexpression delays neurodegeneration and prolongs survival in amyotrophic lateral sclerosis mice. *J Neurosci* 27, 304-307.

Weishaupt, J.H., Bartels, C., Polking, E., Dietrich, J., Rohde, G., Poeggeler, B., Mertens, N., Sperling, S., Bohn, M., Huther, G., Schneider, A., Bach, A., Siren, A.L., Hardeland, R., Bahr, M., Nave, K.A., and Ehrenreich, H. (2006). Reduced oxidative damage in ALS by high-dose enteral melatonin treatment. *J Pineal Res* 41, 313-323.

West, M., Mhatre, M., Ceballos, A., Floyd, R.A., Grammas, P., Gabbita, S.P., Hamdheydari, L., Mai, T., Mou, S., Pye, Q.N., Stewart, C., West, S., Williamson, K.S., Zemlan, F., and Hensley, K. (2004). The arachidonic acid 5-lipoxygenase inhibitor nordihydroguaiaretic acid inhibits tumor necrosis factor alpha activation of microglia and extends survival of G93A-SOD1 transgenic mice. *J Neurochem* 91, 133-143.

Weydt, P., Hong, S., Witting, A., Moller, T., Stella, N., and Kliot, M. (2005). Cannabinol delays symptom onset in SOD1 (G93A) transgenic mice without affecting survival. *Amyotroph Lateral Scler Other Motor Neuron Disord* 6, 182-184.

Willing, A.E., Garbuzova-Davis, S., Saporta, S., Milliken, M., Cahill, D.W., and Sanberg, P.R. (2001). hNT neurons delay onset of motor deficits in a model of amyotrophic lateral sclerosis. *Brain Res Bull* 56, 525-530.

Wu, A.S., Kiaei, M., Aguirre, N., Crow, J.P., Calingasan, N.Y., Browne, S.E., and Beal, M.F. (2003). Iron porphyrin treatment extends survival in a transgenic animal model of amyotrophic lateral sclerosis. *J Neurochem* 85, 142-150.

Wu, R., Wang, H., Xia, X., Zhou, H., Liu, C., Castro, M., and Xu, Z. (2009). Nerve injection of viral vectors efficiently transfers transgenes into motor neurons and delivers RNAi therapy against ALS. *Antioxid Redox Signal* 11, 1523-1534.

Xie, Y., Zhou, B., Lin, M.Y., Wang, S., Foust, K.D., and Sheng, Z.H. (2015). Endolysosomal Deficits Augment Mitochondria Pathology in Spinal Motor Neurons of Asymptomatic fALS Mice. *Neuron* 87, 355-370.

Xu, Z., Chen, S., Li, X., Luo, G., Li, L., and Le, W. (2006). Neuroprotective effects of (-)-epigallocatechin-3-gallate in a transgenic mouse model of amyotrophic lateral sclerosis. *Neurochem Res* 31, 1263-1269.

Yan, J., Xu, L., Welsh, A.M., Chen, D., Hazel, T., Johe, K., and Koliatsos, V.E. (2006). Combined immunosuppressive agents or CD4 antibodies prolong survival of human neural stem cell grafts

and improve disease outcomes in amyotrophic lateral sclerosis transgenic mice. *Stem Cells* 24**,** 1976-1985.

Yang, E.J., Jiang, J.H., Lee, S.M., Yang, S.C., Hwang, H.S., Lee, M.S., and Choi, S.M. (2010). Bee venom attenuates neuroinflammatory events and extends survival in amyotrophic lateral sclerosis models. *J Neuroinflammation* 7**,** 69.

Yang, E.J., Kim, S.H., Yang, S.C., Lee, S.M., and Choi, S.M. (2011). Melittin restores proteasome function in an animal model of ALS. *J Neuroinflammation* 8**,** 69.

Yang, Y.S., Harel, N.Y., and Strittmatter, S.M. (2009). Reticulon-4A (Nogo-A) redistributes protein disulfide isomerase to protect mice from SOD1-dependent amyotrophic lateral sclerosis. *J Neurosci* 29**,** 13850-13859.

Zhang, X., Chen, S., Li, L., Wang, Q., and Le, W. (2008). Folic acid protects motor neurons against the increased homocysteine, inflammation and apoptosis in SOD1 G93A transgenic mice. *Neuropharmacology* 54**,** 1112-1119.

Zhang, X., Chen, S., Song, L., Tang, Y., Shen, Y., Jia, L., and Le, W. (2014). MTOR-independent, autophagic enhancer trehalose prolongs motor neuron survival and ameliorates the autophagic flux defect in a mouse model of amyotrophic lateral sclerosis. *Autophagy* 10**,** 588-602.

Zhang, X., Li, L., Chen, S., Yang, D., Wang, Y., Zhang, X., Wang, Z., and Le, W. (2011). Rapamycin treatment augments motor neuron degeneration in SOD1(G93A) mouse model of amyotrophic lateral sclerosis. *Autophagy* 7**,** 412-425.

Zhang, Y., Cook, A., Kim, J., Baranov, S.V., Jiang, J., Smith, K., Cormier, K., Bennett, E., Browser, R.P., Day, A.L., Carlisle, D.L., Ferrante, R.J., Wang, X., and Friedlander, R.M. (2013). Melatonin inhibits the caspase-1/cytochrome c/caspase-3 cell death pathway, inhibits MT1 receptor loss and delays disease progression in a mouse model of amyotrophic lateral sclerosis. *Neurobiol Dis* 55**,** 26-35.

Zhao, C.P., Zhang, C., Wang, Y.H., Zhou, S.N., Zhou, C., Li, W.Y., and Yu, M.J. (2007). Signals in pathological CNS extracts of ALS mice promote hMSCs neurogenic differentiation in vitro. *Cell Biol Int* 31**,** 1428-1435.

Zhao, W., Varghese, M., Vempati, P., Dzhun, A., Cheng, A., Wang, J., Lange, D., Bilski, A., Faravelli, I., and Pasinetti, G.M. (2012). Caprylic triglyceride as a novel therapeutic approach to effectively improve the performance and attenuate the symptoms due to the motor neuron loss in ALS disease. *PLoS One* 7**,** e49191.

Zhao, W., Varghese, M., Yemul, S., Pan, Y., Cheng, A., Marano, P., Hassan, S., Vempati, P., Chen, F., Qian, X., and Pasinetti, G.M. (2011). Peroxisome proliferator activator receptor gamma coactivator-1alpha (PGC-1alpha) improves motor performance and survival in a mouse model of amyotrophic lateral sclerosis. *Mol Neurodegener* 6**,** 51.

Zhao, Z., Lange, D.J., Voustianiouk, A., Macgrogan, D., Ho, L., Suh, J., Humala, N., Thiyagarajan, M., Wang, J., and Pasinetti, G.M. (2006). A ketogenic diet as a potential novel therapeutic intervention in amyotrophic lateral sclerosis. *BMC Neurosci* 7**,** 29.

Zhao, Z., Sui, Y., Gao, W., Cai, B., and Fan, D. (2015). Effects of diet on adenosine monophosphate-activated protein kinase activity and disease progression in an amyotrophic lateral sclerosis model. *J Int Med Res* 43**,** 67-79.

Zheng, C., Nennesmo, I., Fadeel, B., and Henter, J.I. (2004). Vascular endothelial growth factor prolongs survival in a transgenic mouse model of ALS. *Ann Neurol* 56**,** 564-567.

Zhou, C., Zhang, C., Zhao, R., Chi, S., Ge, P., and Zhang, C. (2013). Human marrow stromal cells reduce microglial activation to protect motor neurons in a transgenic mouse model of amyotrophic lateral sclerosis. *J Neuroinflammation* 10**,** 52.

Zhu, S., Stavrovskaya, I.G., Drozda, M., Kim, B.Y., Ona, V., Li, M., Sarang, S., Liu, A.S., Hartley, D.M., Wu, D.C., Gullans, S., Ferrante, R.J., Przedborski, S., Kristal, B.S., and Friedlander,

R.M. (2002). Minocycline inhibits cytochrome c release and delays progression of amyotrophic lateral sclerosis in mice. *Nature* 417**,** 74-78.
